# Noncanonical formation of SNX5 gene-derived circular RNA regulates cancer growth

**DOI:** 10.1038/s41419-024-06980-4

**Published:** 2024-08-18

**Authors:** Yi-Tung Chen, Hui-Ju Tsai, Chia-Hua Kan, Chung-Pei Ma, Hui-Wen Chen, Ian Yi-Feng Chang, Hsuan Liu, Chih-Ching Wu, Wei-Yun Chu, Ya-Chun Wu, Kai-Ping Chang, Jau-Song Yu, Bertrand Chin-Ming Tan

**Affiliations:** 1 Molecular Medicine Research Center, Chang Gung University, Taoyuan, 333 Taiwan; 2 Department of Biomedical Sciences, College of Medicine, Chang Gung University, Taoyuan, 333 Taiwan; 3 Department of Medical Biotechnology and Laboratory Science, College of Medicine, Chang Gung University, Taoyuan, 333 Taiwan; 4 Graduate Institute of Biomedical Sciences, College of Medicine, Chang Gung University, Taoyuan, 333 Taiwan; 5 Department of Neurosurgery, Lin-Kou Medical Center, Chang Gung Memorial Hospital, Taoyuan, 333 Taiwan; 6 Genomic Medicine Core Laboratory, Chang Gung Memorial Hospital, Taoyuan, 333 Taiwan; 7 Department of Cell and Molecular Biology, College of Medicine, Chang Gung University, Taoyuan, 333 Taiwan; 8 Division of Colon and Rectal Surgery, Lin-Kou Medical Center, Chang Gung Memorial Hospital, Taoyuan, 333 Taiwan; 9 Asia American International Academy, New Taipei City, Taiwan; 10 Department of Otolaryngology-Head & Neck Surgery, Lin-Kou Medical Center, Chang Gung Memorial Hospital, Taoyuan, 333 Taiwan; 11 Research Center for Emerging Viral Infections, Chang Gung University, Taoyuan, 333 Taiwan

**Keywords:** Cell biology, Cancer, Cell growth

## Abstract

Oral squamous cell carcinoma (OSCC) is a prevalent cancer worldwide, exhibiting unique regional prevalence. Despite advancements in diagnostics and therapy, the 5-year survival rate for patients has seen limited improvement. A deeper understanding of OSCC pathogenesis, especially its molecular underpinnings, is essential for improving detection, prevention, and treatment. In this context, noncoding RNAs, such as circular RNAs (circRNAs), have gained recognition as crucial regulators and potential biomarkers in OSCC progression. Our study highlights the discovery of previously uncharacterized circRNAs, including a SNX5 gene-derived circRNA, circSNX5, through deep sequencing of OSCC patient tissue transcriptomes. We established circSNX5’s tumor-specific expression and its strong correlation with patient survival using structure-specific and quantitative PCR analyses. In vitro and in vivo experiments underscored circSNX5 RNA’s regulatory role in cancer growth and metastasis. Further, our omics profiling and functional assays revealed that ADAM10 is a critical effector in circSNX5-mediated cancer progression, with circSNX5 maintaining ADAM10 expression by sponging miR-323. This novel circRNA-miRNA-mRNA regulatory axis significantly contributes to oral cancer progression and malignancy. Moreover, we discovered that circSNX5 RNA is produced via noncanonical sequential back-splicing of pre-mRNA, a process negatively regulated by the RNA-binding protein STAU1. This finding adds a new dimension to our understanding of exonic circRNA biogenesis in the eukaryotic transcriptome. Collectively, our findings offer a detailed mechanistic dissection and functional interpretation of a novel circRNA, shedding light on the role of the noncoding transcriptome in cancer biology and potentially paving the way for innovative therapeutic strategies.

## Introduction

Oral cancer, increasingly recognized as a major global health concern, is one of the most prevalent cancers worldwide. Oral squamous cell carcinoma (OSCC), the most common subtype, accounts for over 90% of all oral cancer cases. Known risk behaviors include betel nut chewing, cigarette smoking, alcohol abuse, and human papillomavirus infection [1–3]. Despite advances in diagnostic and therapeutic strategies, the 5-year survival rate for OSCC patients remains disappointingly stagnant, largely due to aggressive local invasion, distant metastasis, and recurrence [4]. Recent research has highlighted potential biomarkers for oral cancer, such as carcinoembryonic antigen, cancer antigen 19-9, and interleukins (IL)-6 and IL-8 [5, 6]. However, the sensitivity and specificity of these markers are insufficient for effective diagnosis across all oral tumors. This underscores the pressing need for a more profound understanding of OSCC pathogenesis, which could unveil new therapeutic avenues.

Previous studies have unearthed deregulated oncogenes, tumor suppressors, histone modification, and inflammation pathways as the molecular basis of tumorigenesis [7, 8]. While the role of protein-encoded oncogenes or tumor-suppressor genes in OSCC pathogenesis has been extensively studied, the impact of an altered noncoding transcriptome, especially circular RNAs (circRNAs), is yet to be fully unraveled. Thanks to deep sequencing technologies, these once obscure transcriptomes have come to the forefront as crucial gene regulators, despite their lack of protein-coding potential [9, 10]. CircRNAs, characterized by their covalently closed loop structure devoid of a 5’ cap or a 3’-poly (A) tail, exhibit unique biogenesis involving a back-splicing reaction. Their tissue-specific expression patterns and structural stability render promising candidates as biomarkers or therapeutic targets [11, 12].

CircRNAs mediate gene regulation through various mechanisms, such as miRNA sponging and protein scaffold formation. They are predominantly known as competing endogenous RNAs (ceRNAs) that bind target miRNAs, thus liberating mRNA transcripts with shared cognate miRNA-targeted sequences [13]. One of the well-known sponging circRNAs, ciRS-7, contains more than 70 conserved binding sites for miR-7. The interplay between ciRS-7 and miR-7 consequently de-represses EGFR and RAF1 oncogenes in colorectal cancer [14]. CircUHRF1, an oral cancer-associated circRNA, sponges miR-526b-5p to positively regulate MYC protein expression to promote oral cancer progression [15]. Beyond their ceRNA function, circRNAs also regulate gene transcription by trapping RNA-binding proteins (RBPs), as evidenced by the interaction of FMRP protein with circZKSCAN1 RNA, which hinders its transcriptional activity on Wnt signaling and consequently hinders liver cancer progression [16]. Similarly, circRNAs can decoy proteins to modulate protein translation. For instance, circPABPN1 sequesters ELAVL1 protein for the downregulation of its parental PABPN1 mRNA translation [17]; and circANRIL binding to the PES1 protein impedes ribosome biogenesis, leading to nucleolar stress and concomitant p53 activation [18]. Collectively, these findings illustrate the critical involvement of circRNAs in gene expression regulation and its biological and pathogenic consequences.

Despite the growing evidence of circRNAs’ roles in tumorigenesis and their distinct regulatory activities, research on these enigmatic molecules is still in its infancy, and only a few extensively studied [19–21]. Our circRNome profile has previously identified several differentially expressed circRNAs in OSCC, including the oncogenic circFLNB [22]. However, most candidates remain unexplored. The current study focused on circSNX5, which is significantly overexpressed in tumor tissues and correlated with clinical outcomes. Systematic biochemical and functional assays were conducted to elucidate its molecular attributes and oncogenic potential. We discovered that circSNX5 mediates oral cancer progression and malignancy through a novel regulatory signaling involving ADAM10 and miR-323a-5p: circSNX5 was found to sponge miR-323a-5p and maintain ADAM10 expression. Additionally, our work revealed the intricate process of circSNX5 biogenesis via sequential back-splicing, regulated negatively by the RNA-binding protein STAU1. These findings not only illuminate the complexity and diversity of the transcriptome in OSCC but also provide pivotal insights into the circRNA’s contribution to cancer development and progression, potentially paving the way for innovative therapeutic strategies.

## Materials and methods

### Cell culture

SAS and HeLa cells were cultured in high-glucose Dulbecco’s modified Eagle’s medium; SCC25 cells were cultured in Dulbecco’s Modified Eagle Medium: Nutrient Mixture F-12 containing 1× NEAA, 1 mM sodium pyruvate, and 400 ng/mL hydrocortisone. All culture media were supplemented with 10% heat-inactivated fetal bovine serum and 1 U/mL penicillin-streptomycin. All media and reagents were purchased from Thermo Fisher Scientific. These cells were incubated at 37 °C with 5% CO_2_ in a humidified incubator.

### RNA extraction, reverse transcription, and quantitative PCR (RT-qPCR)

Total RNA was isolated by TRIzol reagent (Thermo Fisher Scientific), and complementary DNA (cDNA) was generated by the MML-V reverse transcriptase (Invitrogen) with random hexamers. Individual gene expression was analyzed by real-time quantitative PCR (iQ5 Gradient Real Time SYBR-Green PCR system) with the specific primers and analyzed by CFX Manager Software (Bio-Rad, CA, USA). The sequences of used primer were listed in Supplemental Table S1. The relative gene expression was determined and calculated by double delta Ct method, and the results were obtained from at least three independent experiments. The quantification of PCR amplicons and the signals of immunoblots were assessed by GelQuantNet software. Statistical significance for experiments was evaluated by the Student’s *t* test analysis, and presented in *p* value form in the figures: ns *P* > 0.05; *P < 0.05; **P < 0.01; ***P < 0.001.

### Subcellular fractionation experiment

Subcellular fractionation was performed based on previous report [23]. Briefly, cells were collected and centrifuged into pellet at 4 °C. Nuclear fractionation buffer, containing 10 mM Tris-HCl (pH 7.8), 140 mM NaCl, 1.5 mM MgCl_2_, 0.5% NP-40, 3 U/ml RNaseOUT, was added to the cell pellet and incubated for 5 minutes at 4 °C. Suspended lysate was subsequently centrifuged at 1,000 x g for 4 minutes at 4 °C. The separated supernatant and pellet were collected, and served as the cytoplasmic and nuclear fractions respectively. TRIzol reagent was applied for the RNA extraction of fractions, and subsequent cDNA synthesis was performed by reverse transcription. U48 and 7SL genes served as the nuclear and cytoplasmic markers, and whose expression was detected as references.

### MTT cell proliferation assay and colony formation assay

MTT cell proliferation assay was performed based on the mitochondrial dehydrogenase activity of the living cells that metabolized MTT substrates. Briefly, 2.5 × 10^4^ cells were seeded in a 24-well culture plate and incubated with MTT reagent (Sigma) for 1 hour, and the formed precipitates were dissolved and quantified by spectrophotometry at 570 nm for determining the cell viability. For the colony formation assay, 2.5 × 10^3^ cells were seeded in a 6-well plate for a 7-day culture, and the forming colonies were stained by crystal violet, and quantified by ImageJ software.

### Wound healing assay and transwell experiments

For wound healing assay, 1.5 × 10^6^ cells were seeded in 6-well plate and subsequently scratched by a pipette tip. Wounded cell migration at the indicated time-points were recorded via the Cytation^TM^ 5 Cell Imaging instrument. Transwell migration and invasion assays were performed with Transwell polystyrene membrane Insert (Corning) and Matrigel (BD Biosciences). Briefly, 1.0 × 10^5^ cells were seeded into the Transwell chamber coated with Matrigel (invasion) or without (for migration); serum-free medium was added to the top chambers, and the lower level was filled with culture medium. After 16 h incubation, the migrating (invading) cells through chambers were fixed, stained, and counted.

### Mouse xenograft experiment

NOD.CB17-Prkdc^scid^/JNarl male mice (6 weeks old) were provided by the National Laboratory Animal Center (NLAC). Specific cell lines (10^6^ cells) were collected and inoculated subcutaneously into rear flank of mice with 26-gauge needle. Tumor formation and growth curves were monitored by Vernier caliper at indicated time-points, and tumor volumes (mm^3^) were calculated with formula: length × width^2^ × 0.52. Tumor weight was measured after mice sacrifice. The animal experiments were approved by Laboratory Animal Center, Chang Gung University (CGU110-098).

### Gene knockdown and overexpression by plasmids and synthetic nucleic acid

RNAi-mediated gene silencing was performed by using the pLKO RNAi system, with the target sequences annealed and ligated into vectors. For constructing the circSNX5 expression vector, the exonic region of circSNX5 RNA and the flanking Alu elements were amplified from the cDNA sample. The resulting PCR products were ligated into the cloning vector using the HE Swift Cloning Kit (BIOTOOLS), and subsequently sub-cloned into the expression vector pcDNA3.1 (−) and lentiviral-specific pLAS3W vector. The site-directed mutagenesis was accomplished by PCR reaction within the specific primers. Viral package of the constructs and infection were conducted based on manufacturer’s instructions. Synthesized siRNA duplexes and miRNA mimics purchased from MDBio and GeneDirex respectively, and that were delivered into cells via Lipofectamine RNAiMAX reagent (Thermo Fisher Scientific) with the procedures referring to the Guidelines for Transfection. All used primers in this work were listed in Supplemental Table S1.

### RNA-immunoprecipitation (RNA-IP) assay

RNA-IP experiment was performed based on the previous report [24]. In brief, cells were harvested and washed with PBS, and then lysed by RNA-IP lysis buffer, containing 100 mM KCl, 5 mM MgCl2, 10 mM HEPES (pH 7.0), 0.5% NP40, 1 mM DTT, 50 U/ml RNaseOUT, and protease inhibitor (Roche). Lysates were centrifuged at 12,000 x g for 15 min at 4°C, and the supernatant was collected and incubated with Dynabeads Protein G (Invitrogen) for 1 hour at 4 °C. The pre-cleared lysates were incubated with control IgG or antibody coated Dynabeads for 3 hour at 4 °C. The precipitated complex was washed twice, and treated with DNase I. TRIzol reagent was added for RNA extraction. Purified RNA was transcribed to cDNA, and that was subjected to RT-qPCR analysis.

### pMIR-REPORT luciferase reporter assay

Reporter constructs were generated by subcloning the amplified 3’ UTR of ADAM10 gene into the pMIR-REPORT luciferase vector. Reporter plasmids, Renilla-encoding vector, miR-323 mimics, and circSNX5 expression construct were delivered into cells, and the transfected cells were collected and subjected to Dual-Luciferase® Reporter Assay System (Promega). Luciferase intensity was normalized to the Renilla reads to acquire the relative luciferase activity.

### Antibodies for western blot assays

The used primary antibodies in Western blotting were listed as follows: PARP antibody (sc-7150, Santa Cruz Biotechnology), GAPDH antibody (mAb#2118, Cell Signaling Technology), ADAM10 antibody (ab124695, Abcam), control rabbit IgG (P120-101, Bethyl), and STAU1 antibody (ab73478, Abcam). Secondary antibodies for immunoblotting were purchased from Jackson ImmunoResearch.

### Ethics approval and consent to participate

This study was approved by the Institutional Review Board of Chang Gung Memorial Hospital as a retrospective analysis (201700774B0C501) and was conducted in accordance with the guidelines of the Declaration of Helsinki. Patients were informed about the study design, and all participants provided written informed consent to participate.

## Results

### Transcriptomic profiling uncovers the cancer-associated expression of circSNX5 RNA in OSCC

**Fig. 1:**
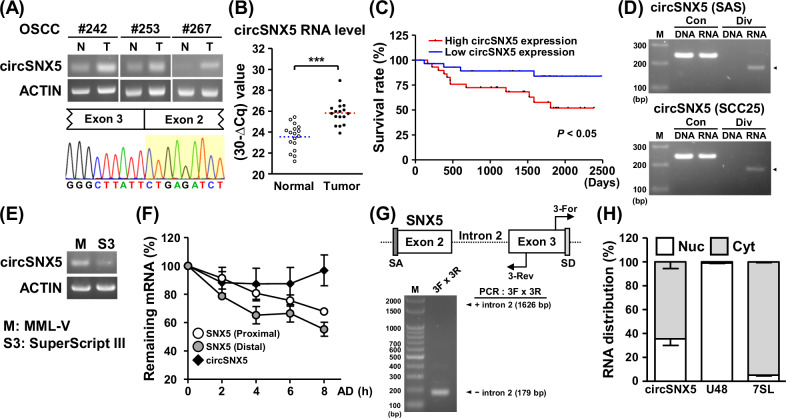
Tumor-biased circSNX5 expression associates with patient survival. **A** CircSNX5 RNA expression of paired OSCC samples was analyzed by end-point PCR assays, and PCR amplicons were analyzed by Sanger method to visualize back-splicing events. ACTIN expression served as the internal control. **B** CircSXN5 expression in normal and tumor tissues was analyzed by RT-qPCR assays, and TBP gene expression served as the internal control. **C** Kaplan-Meier survival analysis of the patients was performed in correspondence to the circSNX5 expression (*n* = 118). **D** PCR assays of genomic DNA (DNA) and transcribed cDNA (RNA) of SAS cells were performed with convergent (Con) and divergent (Div) primers, and the amplicons were analyzed by gel electrophoresis. **E** PCR analyses of cDNA synthesized by M-MLV or SuperScript III reverse transcriptases from SAS cells were performed and visualized by gel electrophoresis. ACTIN expression served as the loading control. **F** SAS cells were treated with actinomycin D (AD) for the indicated time lengths, and harvested for RT-qPCR analyses. RNA turnover rate was measured by normalizing to the abundance at initial time point and plotted. **G** PCR assays with specific primers were performed to analyze structural organization of circSNX5 RNA derived from SAS cells, and PCR products were analyzed by gel electrophoresis. Putative splicing acceptor and donor sites were denoted as SA and SD. **H** Subcellular distribution of circSNX5 RNA of SAS cells was determined by RT-qPCR assays of fractionation experiment, and the marker genes were analyzed (nucleus: U48, cytosol: 7SL).

Previously, our analysis identified dysregulated circRNAs in OSCC specimens [22, 25], among which a circular RNA derived from the SNX5 gene (circSNX5) was significantly upregulated in tumor tissues compared to normal tissues (Fig. 1A, upper panel; Fig. S1A). This noticeable difference led to its selection for further investigation. The sequencing chromatograms of PCR amplicons confirmed the ‘head-to-tail’ splice junction of the circSNX5 transcript, demonstrating specific back-splicing events (Fig. 1A, lower panel). Additionally, RT-qPCR assays conducted on samples from another cohort revealed the enhanced circSNX5 RNA expression in tumor tissues (Fig. 1B), corroborating our initial findings. Incidentally, the circSNX5 expression was also detected in oral cancer cell lines (Fig. S1B). Kaplan–Meier survival analysis further indicated a correlation between higher circSNX5 expression and poorer survival rate, suggesting its potential role in clinical outcomes (Fig. 1C). These findings revealed the association of circSNX5 upregulation with oral cancer development and its clinical relevance, which prompted us to select circSNX5 for advanced characterization and functional studies.

To characterize the molecular attributes of circSNX5 RNA, we conducted convergent and divergent PCR assays. These assays specifically amplified the synthesized complementary DNA (cDNA) rather than genomic DNA, confirming that circSNX5 arises from transcriptional processing rather than gene recombination (Figs. 1D and S1C). PCR amplification using templates from MML-V and SuperScript transcriptases showed the consistent results (Fig. 1E), ruling out false-positive junction formation in cDNA synthesis [26]. The structural circularity of circRNAs, known for their resistance to exonuclease-mediated degradation, enhances their stability. Monitoring the turnover rate of circSNX5 and its parental linear mRNA under actinomycin D treatment revealed a significant reduction in linear transcript levels after 8 hours, whereas circSNX5 levels remained stable (Figs. 1F and S1D). This indicates the enhanced stability of the alternative-splicing SNX5 product, the circSNX5 transcript, in cells.

Further exploration of circSNX5’s molecular attributes led us to investigate its exon–intron organization and subcellular localization. Using specific primers for the circularized exons or adjoining introns, we determined the sequence arrangement of the circSNX5 molecule (Fig. 1G, upper panel). Gel electrophoresis confirmed the absence of introns, classifying circSNX5 as an exonic circRNA derived from SNX5 exons 2 to 3 (Fig. 1G, lower panel). Subcellular fractionation assays revealed the presence of circSNX5 in both cytosolic and nuclear compartments (Fig. 1H, lane 1). with U48 and 7SL RNA serving as nuclear and cytosolic markers, respectively (Fig. 1H, lanes 2 and 3). Considering the potential of some circRNAs to associate with translating ribosomes and produce small peptides [27], we isolated the ribosome-nascent chain complex (RNC) to assess translatability of circSNX5 RNA. RT-qPCR analysis detected negligible amounts of circSNX5 in the RNC–mRNA fraction, suggesting low translatability of this transcript (Fig. S1E), in contrast to the abundant detection of ACTIN mRNA, a known translating gene, in the same fraction.

### CircSNX5 knockdown compromises in vitro and in vivo tumor attributes

**Fig. 2:**
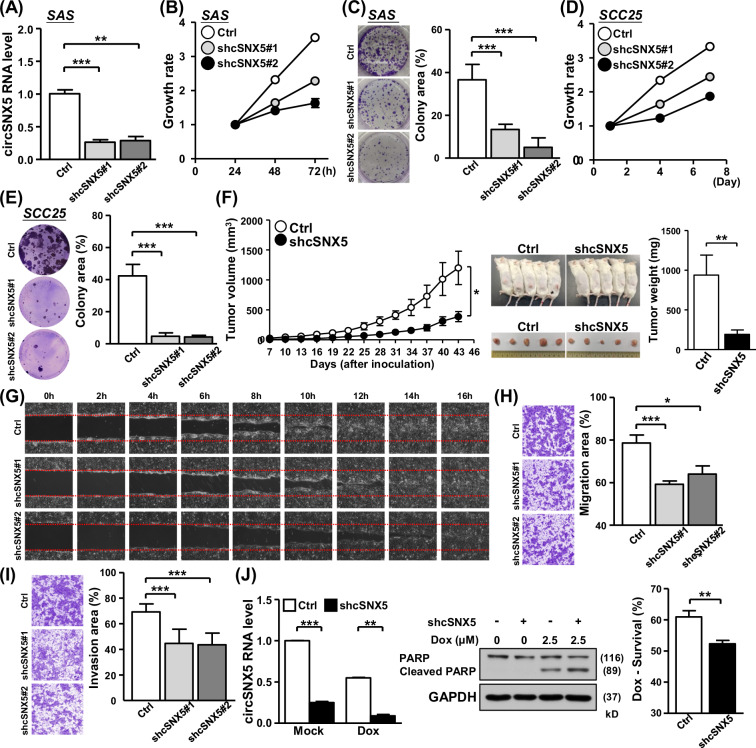
CircSNX5 suppression compresses cancer-associated phenotypes. **A**–**E** CircSNX5 knockdown of oral cancer lines SAS and SCC25 were conducted (shcSNX5#1 and shcSNX5#2), and knockdown efficacy in SAS cells was evaluated by RT-qPCR assay (**A**). Cell proliferation (**B**, **D**) and clonogenicity (**C**, **E**) of the cells were assessed by MTT method and crystal violet staining, respectively. Colony images and quantified results of indicated cells were shown. **F** The mouse xenograft experiment was performed by inoculating control and circSNX5 knockdown cells. Tumors formed at the indicated time points were dissected and measured for the volume in the left panel. The photographs of mice bearing tumors and the weight of resected tumors were showed. **G** The wound healing migration assay of SAS cells were performed, and representative images of the indicated time points after scratching were showed. **H**, **I** Transwell migration and Matrigel invasion assays of control and knockdown SAS cells were performed. The representative photographs for indicated groups were shown in left panel, and the relative migration and invasion area were quantified and showed in bar graphs. **J** SAS cells were treated with doxorubicin to induce apoptosis, and circSNX5 RNA expression and PARP protein expression/cleavage of treated cells were analyzed by RT-qPCR and Western blot assays respectively. GAPDH expression served as the loading control of immunoblotting. Cell survival of treated cells were determined by MTT method.

After establishing the biological attributes of the circSNX5 molecule, we focused on deciphering its functional implications in OSCC. We employed shRNAs targeting the back-splicing junction site of circSNX5, introducing them into cells. The efficiency of this knockdown was verified through RT-qPCR experiments (Fig. 2A). In SAS cells, circSNX5 knockdown led to a noticeable reduction in both proliferation rate and colony formation ability compared to control cells (Fig. 2B, C). This finding highlights the critical role of circSNX5 expression in oral cancer growth. A similar pattern was observed in SCC25 cells, where targeting circSNX5 expression resulted in decreased cancer cell proliferation and clonogenicity (Fig. 2D, E; Fig. S2A), further supporting circSNX5 RNA’s pro-growth role in oral cancer cell lines.

Subsequently, circSNX5 knockdown cell lines were assessed in vivo for tumor formation. The results recapitulated those of the cell-based assays, with the circSNX5 knockdown group exhibiting significantly smaller tumor volumes (Fig. 2F, left panel) and tumor weights (Fig. 2F, right panel) compared to the control group. This concordance underscores circSNX5’s importance in oral cancer growth. Interestingly, parental SNX5 expression remained unaffected under circSNX5 knockdown conditions (Fig. S2B), suggesting an independent regulation mechanism for circSNX5 RNA alternative splicing from the parental SNX5 gene. This finding excludes the possibility of mutual regulation between the linear SNX5 gene and its circular variant [28].

Exploring further, we examined circSNX5 RNA’s regulatory role in cancer cell metastasis and survival. Wound healing assays revealed the delayed cell migration in circSNX5 knockdown conditions (Fig. 2G), while Transwell migration and invasion assays affirmed the significance of circSNX5 in OSCC metastatic properties (Fig. 2H, I). These results link circSNX5 expression to the aggressive features of tumor cells. Additionally, we investigated circSNX5’s association with the response to anti-cancer drugs. In the presence of doxorubicin, circSNX5 knockdown cells exhibited markedly enhanced PARP cleavage, indicative of increased apoptotic activity, along with reduced cell viability (Fig. 2J). In parallel, circSNX5 knockdown also heightened the sensitivity of cancer cells to cisplatin (Fig. S3). These findings suggest that targeting circSNX5 could potentially sensitize cells to anticancer treatments, proposing circSNX5 as a positive regulator in cancer cell growth and survival and further a valuable therapeutic target in cancer treatment.

### CircSNX5 RNA is generated by a noncanonical back-splicing mechanism

**Fig. 3:**
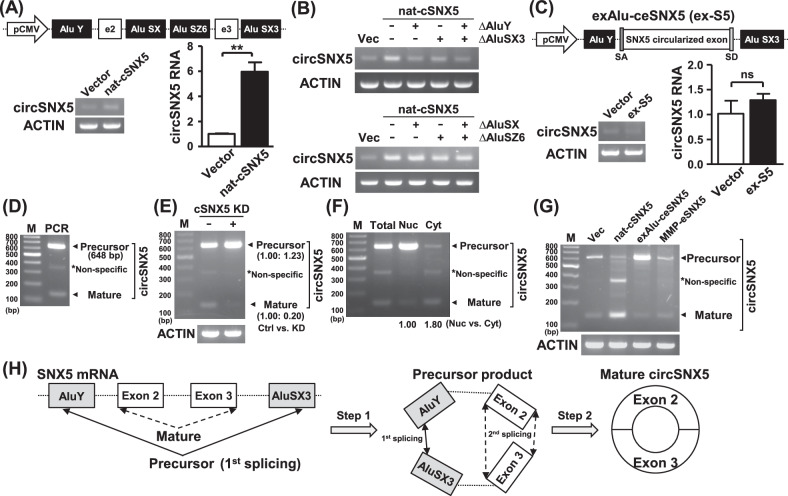
CircSNX5 biogenesis operates by the sequential back-splicing mechanism. **A** The circSNX5 vector (nat-cSNX5) was constructed and transfected into cells, and the expression efficiency was analyzed by end-point PCR and RT-qPCR assays. ACTIN expression served the loading control. **B** The mutant circSNX5 constructs were generated and delivered into cells, and the circSNX5 expression of transfectants was assessed by PCR assays. ACTIN expression served as internal control. **C** The circSNX5 construct (exAlu-ceSNX5) was transfected into cells, and the circSNX5 expression of transfectants was analyzed by end-point PCR and RT-qPCR analyses. ACTIN expression was used as the loading control. **D** PCR assays with increasing extension were performed, and the resulting products were visualized by gel electrophoresis. PCR amplicons were noted by the arrows while the asterisk (*) denotes non-specific signal. **E** CircSNX5 PCR analyses of indicated cells were performed, and ACTIN expression served the loading control. PCR products were analyzed by gel electrophoresis and further quantified. **F** CircSNX5 PCR assays of indicated samples were performed and analyzed by gel electrophoresis. PCR amplicons were noted by arrows and the asterisk. **G** CircSNX5 PCR analyses of transfectants were performed and analyzed by gel electrophoresis. ACTIN expression served the loading control. **H** Proposed model for the sequential back-splicing mechanism of circSNX5 biogenesis.

For the construction of the circSNX5 expression vector, we utilized the UCSC genome browser to identify potential repeats for back-splicing formation (Fig. S4A). Sequence alignment analyses revealed the complementarity of the paired Alu repeats (Fig. S4B), which were probably to induce circSNX5 RNA formation. We then created the “natural” circSNX5 expression vector (nat-cSNX5), containing non-spliced exons and Alu element-embedded intronic sequences for advanced molecular studies (Fig. 3A, upper panel). The expression efficacy of the circSNX5 construct was confirmed through both end-point PCR and RT-qPCR assays (Fig. 3A, lower panel).

Our next goal was to pinpoint the fundamental sequences critical for circRNA formation. Deletion of the external Alu repeats markedly reduced the nat-cSNX5 construct’s capacity to form circRNA (Fig. 3B, upper panel; Fig. S5A), underscoring their necessity. Furthermore, manipulating the AluSX or AluSZ6 repeats individually or in combination did not significantly reduce the nat-cSNX5 plasmid’s expression activity (Fig. 3B, lower panel; Fig. S5B), indicating that circSNX5 formation relies on external Alu repeats rather than internal ones. Additionally, we explored the role of the polypyrimidine tract and splicing factors in this process [29] However, deletion of the polypyrimidine tract had a negligible effect on expression activity (Fig. S5C), suggesting that circSNX5 formation is regulated independently of this mechanism.

The canonical back-splicing process for exonic circRNAs typically occurs after intron removal, with lateral complementary repeats mediating the back-splicing of ligated exons [30, 31]. To investigate this, we constructed a circSNX5 expression vector (exAlu-ceSNX5) comprised of external Alu elements and exon-joined sequences without intronic sequences (Fig. 3C, upper panel). Surprisingly, this construct failed to activate circSNX5 RNA expression (Fig. 3C, lower panel), suggesting an atypical back-splicing mechanism of the circRNA. This led us to extend the PCR extension time to explore potential transitional products in circSNX5 formation. An unexpected larger PCR product was identified (Fig. 3D), and sequencing of this 648-bp fragment revealed it contained non-spliced intronic sequences and an enigmatic junction of AluY and AluSX3 sequences (Fig. S6A). We hypothesized this product, a cryptic back-splicing product of AluY and AluSX3 repeats, serves as a precursor for noncanonical circSNX5 formation.

In circSNX5 knockdown cells, we observed an increase of precursor circSNX5 RNA expression (Figs. 3E and S6B), suggesting a possible feedback mechanism in circSNX5 processing. Subcellular fractionation assays revealed distinct distributions for precursor and mature forms of circSNX5 RNA, predominantly in the nucleus and cytoplasm, respectively (Figs. 3F and S6C). This finding aligned with the co-transcriptional RNA processing occurring mainly in the nucleus, while spliced transcripts transported to the cytoplasm [32]. Long-term RNA stability analysis showed a decline in precursor circSNX5 RNA expression over time, while the expression of mature circSNX5 RNA remained stable (Fig. S6D), highlighting the transient nature of the precursor form and the stability of mature circSNX5 RNA.

Further, circSNX5 PCR assays using the full-length expression vector (nat-cSNX5 construct) notably enhanced mature circSNX5 expression, while reducing precursor RNA level (Fig. 3G, lane 3). This observation supports the mutual regulation of circSNX5 variants (Fig. 3E). Significant accumulation of the precursor variant in exAlu-ceSNX5 transfection suggested the abrogation of processing of circSNX5 precursor RNA (Fig. 3G, lane 4), and further indicated the occurrence of subsequent back-splicing removed intronic sequences to generate mature circSNX5 RNA. Our recent study identified the enhanced expression of circMMP12 RNA in OSCC tumor tissues (Fig. S7A). The adjacent bilateral Alu repeats of the MMP12 gene were found capable of stimulating circRNA formation (Fig. S7B). To further investigate, we subcloned MMP12-derived Alu sequences into the circularized exon of the BNC2 gene (Fig. S7C, upper panel), and observed that circBNC2 overexpression confirmed the capacity of Alu repeats in circRNA formation (Fig. S7C, lower panel). Creating a specific circSNX5 vector with circulated exons and inverted Alu elements from the MMP12 gene (MMP-eSNX5) (Fig. S7D), we inspected the expression efficiency of this construct. The failure of MMP-eSNX5 to activate circSNX5 expression underlined the importance of precursor formation in circSNX5 biogenesis (Fig. 3G, lane 5; Fig. S7E), and highlighted the unique and enigmatic back-splicing of external Alu repeats on the circSNX5 gene. In summary, our analyses elucidate the essential role of precursor circSNX5 formation by external Alu elements and demonstrate the complex back-splicing mechanism in circSNX5 biogenesis (Fig. 3H).

### CircSNX5 overexpression promotes cancer cell growth and metastasis

**Fig. 4:**
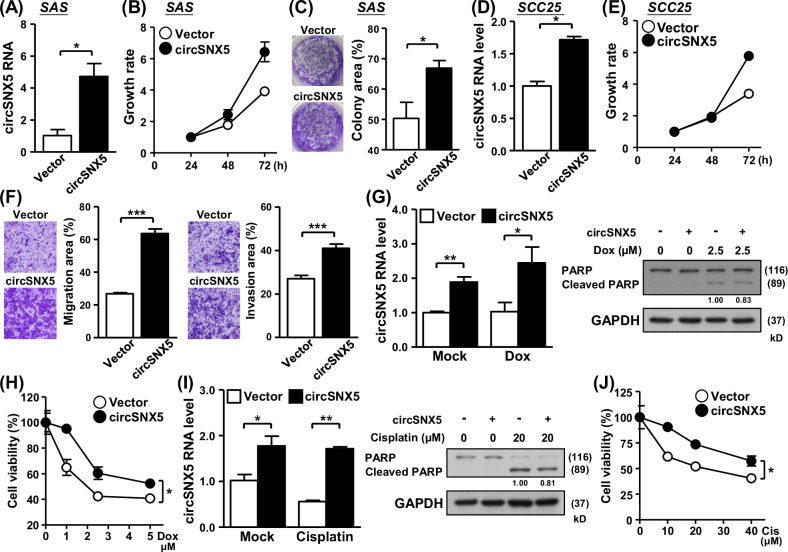
CircSNX5 overexpression promotes cancer growth and metastasis. **A**–**C** CircSNX5 overexpression of SAS cells was conducted, and circSNX5 expression of the cells was analyzed by RT-qPCR assay. Cell proliferation and colony formation ability of cells were assessed by MTT method and crystal violet staining, respectively. Colony images and quantified results were shown. **D**, **E** CircSNX5 overexpression of SCC25 cells was conducted, and the circSNX5 expression and cell proliferation of cells was analyzed by RT-qPCR assay and MTT method respectively. **F** Transwell migration and invasion assays of the indicated SAS cells were performed. The representative photographs for indicated groups were shown in left panel, and relative migration and invasion area were depicted in bar graphs. **G**–**J** Control and circSNX5 overexpression SAS cells were treated with cisplatin or doxorubicin, and treated cells were collected and subjected to RT-qPCR and Western blot assays to analyze circSNX5 RNA expression and PARP protein cleavage. GAPDH expression served the internal control for immunoblotting. Cell survival was measured by MTT method and plotted.

Following the successful construction of a circSNX5 expression vector, we utilized this tool to investigate the impact of circSNX5 upregulation on cancer phenotypes. In SAS cells, circSNX5 overexpression notably accelerated cell proliferation and enhanced colony formation ability (Fig. 4A–C), presenting a stark contrast to the suppressed cancer attributes observed in the knockdown experiments (Fig. 2). Similarly, ectopic expression of circSNX5 RNA in SCC25 cells significantly increased cell proliferation and clonogenicity, underscoring the pivotal role of circSNX5 in regulating cancer cell growth (Fig. 4D, E; Fig. S8A). Moreover, the circSNX5-overexpressing cells exhibited pronounced increases in cell migration and invasion capabilities (Fig. 4F and S8B). This enhancement suggests that circSNX5 upregulation is a key factor in promoting oral cancer cell metastasis. Further analysis demonstrated a reduced sensitivity to anti-cancer drugs in cells overexpressing circSNX5 (Fig. 4G–J). This observation is in direct opposition to the results obtained from the knockdown experiment, indicating circSNX5’s potential role in mediating cellular responses to anticancer therapy and adverse environmental conditions. These functional assays, focusing on overexpression, revealed a reversal of cancer phenotypes compared to the knockdown studies. These findings not only establish the definitive molecular role of circSNX5 RNA in oral cancer growth and metastasis but also reinforce our earlier observations of the significant contribution of deregulated circSNX5 RNA to the progression and clinical outcomes of oral cancer (Fig. 1).

### STAU1 protein delivers negatively regulation on circSNX5 formation

**Fig. 5:**
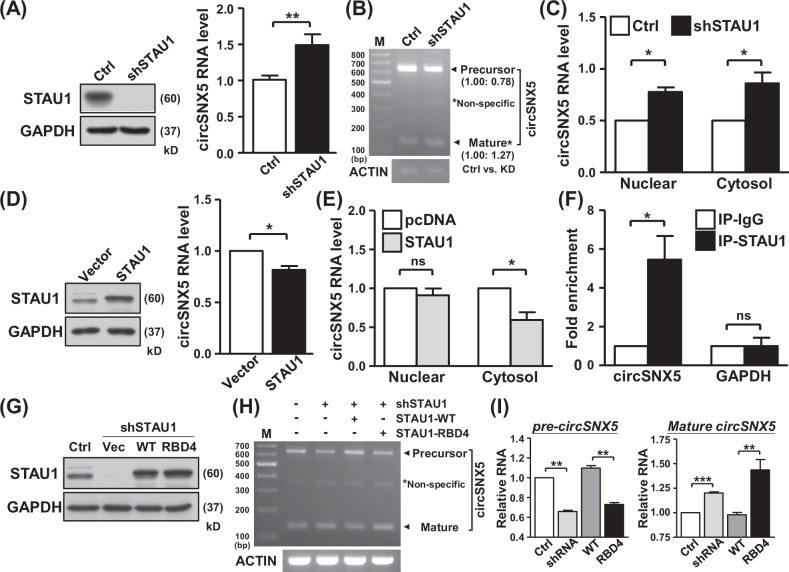
STAU1 protein regulates circSNX5 biogenesis dependent on its RNA-binding activity. **A** Control and STAU1 shRNA were delivered into cells, and the protein and RNA expression of cells was analyzed by Western blot assay and RT-qPCR assay, respectively. GAPDH expression served as internal control for immunoblotting. **B** CircSNX5 PCR assays of indicated cells was performed, and resulting products were visualized by gel electrophoresis. PCR amplicons were annotated and quantified. **C** Subcellular fractionation experiments of indicated cells were performed, and circSNX5 RNA expression of fractions was analyzed by RT-qPCR assay. **D** Protein and RNA expression of control and STAU1 overexpressing cells were measured by Western blot assay and RT-qPCR analysis, respectively. GAPDH expression served as loading control for immunoblotting. **E** CircSNX5 expression of subcellular fractions in control and STAU1 overexpression cells was determined by RT-qPCR assays. **F** RNA-IP assay was performed with control and STAU1-specific antibodies, and precipitates was collected and subjected to RT-qPCR assay with the indicated primers. **G**, **I** Indicated STAU1 plasmids were delivered into the cells, and protein expression of transfectants was analyzed by Western blot assays. GAPDH expression served as internal control for immunoblotting. CircSNX5 PCR assays of the transfectants were performed and visualized by gel electrophoresis (**H**), and the PCR amplicons were noted and quantified.

Given the pivotal role of RNA-binding proteins (RBPs) in dictating the expression and distribution of circRNAs [33, 34], we investigated the regulation of circSNX5 RNA by such mechanisms. Previously, we identified STAU1 protein as a competitor with ADAR1 protein for binding to double-stranded RNA, influencing the editing frequency and distribution of target transcripts [35]. We first assessed ADAR1 and STAU1’s regulation on circSNX5. RT-qPCR analysis of ADAR1 knockdown cells showed no significant change in circSNX5 expression, indicating a minimal effect of ADAR1 protein on circSNX5 (Fig. S9A). However, STAU1 knockdown markedly enhanced circSNX5 RNA expression (Figs. 5A and S9B). Additionally, a decrease in precursor circSNX5 RNA in STAU1-knockdown cells (Figs. 5B and S9C) suggested STAU1’s involvement in the circSNX5 splicing process, leading to the upregulation of mature circSNX5 RNA.

Subcellular fractionation assays revealed increased expression of mature circSNX5 RNA in both nuclear and cytoplasmic compartments following STAU1 knockdown (Fig. 5C). Notably, STAU1 silencing had a more pronounced inhibitory effect on cytoplasmic expression of circSNX5 precursor than in the nuclear fraction (Fig. S9D), implying STAU1’s role in processing precursor circSNX5 and its transportation from the nucleus to the cytoplasm. Conversely, ectopic overexpression of STAU1 inhibited circSNX5 expression (Fig. 5D), with fractionation assays showing the reduced cytoplasmic circSNX5 RNA in STAU1-overexpressing cells (Figs. 5E and S10A). These results from STAU1 overexpression experiments, contrasting with the knockdown findings, confirm STAU1’s regulatory role in circSNX5 formation. Furthermore, RNA immunoprecipitation assay validated the interaction between STAU1 protein and circSNX5 RNA, supporting STAU1’s influence on the alternative splicing of the SNX5 gene (Fig. 5F). These findings collectively demonstrate STAU1’s involvement in fine-tuning circRNA biogenesis through its regulation on splicing and transportation mechanisms in the SNX5 gene.

STAU1 protein is recognized as a key regulator in eukaryotic mRNA processing, distribution, and degradation [36–38], particularly through its binding to inter- and intra-RNA duplexes, mediating the specific degradation of target mRNAs in the STAU1-mediated mRNA decay (SMD) pathway. To explore the potential link between the SMD mechanism and STAU1–circSNX5 regulation, we silenced UPF2, a core component of the SMD mechanism, using siRNA transfection (Fig. S10B, left). The expression of circSNX5 RNA remained consistent following UPF2 knockdown (Fig. S10B, right), suggesting a minimal connection between SMD and circSNX5 regulation. Additionally, RNA stability analyses indicated that while STAU1 knockdown augmented basal circSNX5 RNA expression, it did not alter the turnover rate (Fig. S10C, D), excluding the involvement of the SMD mechanism in STAU1–circSNX5 regulation.

We then employed a knockdown-rescue system using wild-type STAU1 protein and RBD4 domain mutant constructs to survey the STAU1–circSNX5 interaction (Fig. 5G) [39]. Reintroducing wild-type STAU1 in knockdown cells reinstated STAU1’s inhibitory effect on circSNX5 expression (Fig. 5H, lane 4; Fig. S10E), whereas the mutant RBD4 STAU1 protein failed to impact circSNX5 expression (Fig. 5H, lane 5). The expression of precursor circSNX5 RNA inversely correlated with mature circSNX5 RNA levels, in line with the alteration of STAU1’s RNA-binding activity (Fig. 5I) [40]. This supports the hypothesis that STAU1 regulates the circSNX5 splicing mechanism. Taken together, these collaborative findings demonstrate STAU1’s negative regulatory role in the circRNA back-splicing process, leading to the concurrent accumulation of precursor circSNX5 RNA.

### CircSNX5 functions as the miRNA sponge to promote cancer growth

**Fig. 6:**
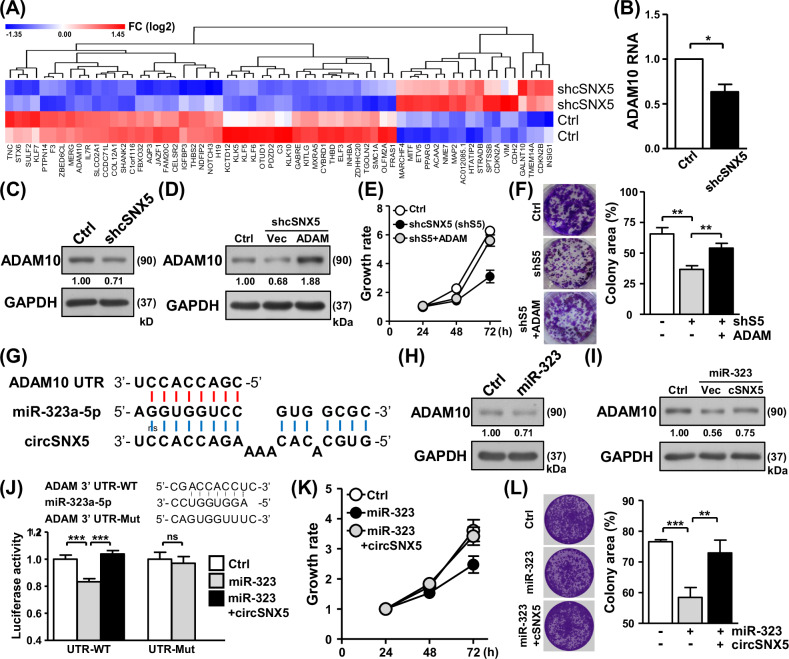
CircSNX5 RNA functions as miRNA sponge to regulate oral cancer progression. **A** Differentially expressed genes in the control versus circSNX5 knockdown comparison were illustrated in a heatmap representation, and the expression value was displayed according in a color scale bar. **B**, **C** The ADAM10 RNA and protein expression of control and knockdown SAS cells were analyzed by RT-qPCR analysis and Western blot assays, respectively. **D**–**F** ADAM10 vector was delivered into SAS knockdown cells, and the transfected cells were collected and subjected to protein expression assay, cell proliferation analysis, and colony formation assay. **G** The putative binding sequences for the proposed circSNX5-mediated miRNA sponge on ADAM10 mRNA were illustrated. **H**, **I** ADAM10 protein expression of the transfectants of SAS cells were analyzed by Western blot assays, GAPDH expression served as the loading control. **J** The reporter constructs were co-transfected with miR-323 mimic and circSNX5 plasmid, and transfected cells were harvested and subjected to luciferase reporter assays. **K**, **L** SAS cell proliferation and colony formation assays of the transfectants were analyzed by MTT method and crystal violet staining respectively. Colony images and quantified results were shown.

These observations underscored the oncogenic potential of circSNX5 in oral cancer. To unravel the underlying mechanisms and characterize circSNX5-mediated molecular regulation, we conducted RNA-sequencing experiments to profile transcriptome alterations following circSNX5 knockdown. A total of 63 genes were identified differentially expressed between the groups (|fold change | ≥ 1.5, *p* value < 0.05), with their overall distribution depicted in a hierarchical heatmap representation (Fig. 6A). Bioinformatics analysis of these genes linked them to cancer pathways, reinforcing circSNX5’s oncogenic influence (Fig. S11A). Incorporating proteomic profiling of the knockdown experiment recognized the downregulation of ADAM10 expression in circSNX5 knockdown cells, as confirmed by RT-qPCR and Western blot assays (Fig. 6B, C; Fig. S11B). Moreover, RT-qPCR analysis of clinical samples revealed co-expression patterns between circSNX5 and ADAM10 transcripts (Fig. S12A), leading us to hypothesize the effector role of ADAM10 in circSNX5-mediated oncogenesis based on its association with cancer [41].

To further investigate the role of ADAM10 in circSNX5 signaling, we performed rescue experiments involving the downregulation of circSNX5 and the ectopic expression of ADAM10. Ectopic expression of ADAM10 alleviated the reduced proliferation and clonogenicity in SAS cells caused by circSNX5 knockdown (Figs. 6D–F), demonstrating the significance of ADAM10 expression in circSNX5 signaling. Specifically, the overexpression of ADAM10 reversed the phenotypic effects of circSNX5 knockdown, restoring cell proliferation and colony formation abilities. Concurrently, rescue experiments in SCC25 cells yielded similar results, confirming our hypothesis (Fig. S12B–D). These experiments showed that the reintroduction of ADAM10 could mitigate the inhibitory effects on cell proliferation and clonogenicity induced by circSNX5 knockdown. Furthermore, xenograft assays with rescue treatments demonstrated the essential role of ADAM10 expression in circSNX5-mediated regulation of in vivo cancer growth (Fig. S12E). In these assays, SAS cells with circSNX5 knockdown and ectopic ADAM10 expression were injected into mice. The results showed that the overexpression of ADAM10 reversed the reduced tumor growth caused by circSNX5 knockdown, highlighting ADAM10’s critical involvement in circSNX5-mediated oncogenesis. These findings illustrated ADAM10’s pivotal role in the circSNX5 signaling pathway, both in vitro and in vivo.

Considering the nature of cytoplasmic circRNAs in mediating gene regulation [42, 43], we performed bioinformatic analyses and found that circSNX5 RNA potentially acts as a miRNA sponge, protecting ADAM10 from degradation by tumor suppressor miR-323a-5p (Fig. 6G) [44]. To test this hypothesis, we introduced a miR-323a-5p mimic and observed its impact on circSNX5 regulation. Western blot assay confirmed that miR-323 expression led to decreased ADAM10 protein levels (Figs. 6H and S13A), validating the postulated miRNA-mRNA regulatory mechanism. Subsequently, circSNX5 overexpression mitigated miR-323-mediated ADAM10 degradation (Fig. 6I; Fig. S13B, C), indicating the protective effect of circSNX5 through a miRNA sponging mechanism.

To further substantiate the direct interaction between circSNX5 and miR-323, we performed RNA pull-down assays using biotinylated circSNX5-specific probes and miR-323 mimics. The circSNX5-specific probes were designed to selectively bind circSNX5 RNA, allowing for the isolation and identification of interacting molecules. Conversely, biotinylated miR-323 mimics were used to capture miR-323 and its interacting RNAs. These pull-down assays revealed a significant enrichment of miR-323 in complexes pulled down by circSNX5-specific probes (Fig. S13D), demonstrating that circSNX5 directly binds to miR-323. Similarly, the pull-down with biotinylated miR-323 mimics showed a substantial capture of circSNX5 RNA, further confirming the direct interaction between these molecules (Fig. S13E). These findings robustly support the molecular activity mediated by circSNX5 RNA, validating its role as a miRNA sponge.

We then employed a luciferase reporter system to further investigate the sequence complementarity of RNA–RNA interactions. The presence of miR-323 reduced the luciferase activity of the wild-type ADAM10 reporter construct (UTR-WT) (Fig. 6J, lanes 1 and 2), while mutations in the putative binding sequence (UTR-Mut) abrogated miR-323 mediated degradation on the luciferase gene (Fig. 6J, right panel). This confirmed that miR-323 targets ADAM10 expression through the recognition of 3’ UTR. Importantly, circSNX5 expression counteracted miR-323’s regulatory effect on reporter activity (Fig. 6J, lane 3), substantiating the circSNX5-ADAM10 regulatory axis via a miRNA sponge mechanism.

To further validate the role of circSNX5 in antagonizing miR-323’s effects, we conducted rescue experiments involving the overexpression of circSNX5. These experiments demonstrated that circSNX5 overexpression reversed the inhibition of cancer growth caused by miR-323 expression. Specifically, in SAS cells, circSNX5 overexpression counteracted the miR-323-mediated suppression of cell proliferation and clonogenicity, restoring these functions to levels comparable to controls (Fig. 6K, L). Parallel rescue experiments in SCC25 cells provided consistent findings, reinforcing our hypothesis (Fig. S14A–C). Furthermore, tumor xenograft experiments illustrated that circSNX5 overexpression mitigated the inhibitory effects of miR-323 on *in vivo cancer* growth (Fig. S14D). In these experiments, SAS cells with miR-323 overexpression and concurrent circSNX5 overexpression were injected into mice. The results showed that the overexpression of circSNX5 reversed the reduced tumor growth induced by miR-323, highlighting circSNX5’s critical role in miRNA-mediated regulation of tumor growth. Additionally, Transwell assays revealed that circSNX5 expression neutralized miR-323-mediated suppression of cell metastasis, thereby promoting cancer cell metastasis (Fig. S15). These results collectively elucidated circSNX5’s role in sponging miR-323 to promote cancer progression. In summary, these findings offer a mechanistic dissection and functional interpretation of the novel circSNX5-miR323-ADAM10 regulatory axis, contributing significantly to cancer progression and malignancy. This highlights the intricate interplay of noncoding RNAs in oral cancer biology and underscores the potential of targeting such mechanisms in therapeutic strategies.

## Discussion

**Fig. 7:**
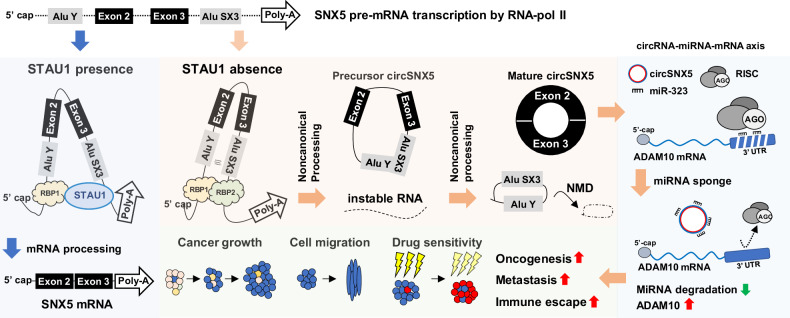
Schematic illustration of circSNX5 biogenesis mechanism and its functional implications. Proposed molecular interplays for circSNX5 back-splicing process and its functional regulation on oral cancer progression and malignancy.

Circular RNAs have been recognized as pivotal molecules in maintaining homeostasis and influencing disease progression, notably in cancer development and malignancy [21, 45, 46]. The dysregulation of circRNAs has emerged as a crucial contributor to these processes. Although the biogenesis of circRNAs is known to be regulated by RNA-binding protein mediated interactions that contribute to the back-splicing mechanism [15, 47], a comprehensive understanding of the mechanistic and functional roles of circRNome in cancer biology is still unfolding. Our findings shed new light on this area, providing novel insights into the noncanonical formation of circSNX5 RNA, which is characterized by sequential back-splicing that generates a precursor product in a unique mechanism. Furthermore, our research identifies the RNA-binding protein STAU1 as a negative regulator of this process, mediated by its RNA-interacting domain. Through omics-based profiling and functional assays, we have elucidated the mediator role of ADAM10 in the circSNX5 regulatory signaling pathway. Notably, circSNX5 appears to maintain ADAM10 expression by antagonizing miRNA activity. These insights offer a significant expansion in our understanding of the molecular intricacies of circRNA biogenesis. They also enhance our understanding of the noncoding genome’s role in cancer biology, as illustrated in Fig. 7. This understanding opens new avenues for exploring the potential of circRNAs in cancer diagnostics and therapeutics, underscoring their importance in the wider context of cancer research.

Canonical circRNA biogenesis typically involves back-splicing of exons and/or primary intronic sequences [30], the exact mechanisms governing the removal of intronic sequences during such splicing events remain largely unexplored. Current study marks a crucial step in elucidating these mechanisms, demonstrating the vital role of precursor production in the processing and formation of mature exonic circSNX5 RNA. This provides a robust and verifiable framework for understanding the biogenesis of exonic circRNAs. In exploring the regulation of circSNX5 by RBPs, which are known to influence transcript subcellular distribution and biological outcomes [35], we focused on the effects of ADAR1 and STAU1. Our results, as illustrated in Fig. S9, showed circSNX5 expression remained unaffected by ADAR1 knockdown. Conversely, STAU1 protein emerges as a key negative regulator, interfering with the back-splicing process of circSNX5, as demonstrated in Fig. 5. This distinct RBP-mediated regulation can be attributed to two main factors: Firstly, the absence of editing sites in the circSNX5 transcript sequences (https://genome.ucsc.edu/) suggests that ADAR1 does not operate deamination modification on circSNX5, thereby not impacting its function. Secondly, the presence of inverted Alu repeats on SNX5 mRNA, which forms the structural precursor of circSNX5, likely facilitates STAU1 binding but not ADAR1, indicating context-dependent and/or gene-specific transcriptional processing. Moreover, mutation on the RBD4 domain of STAU1, disrupts dsRNA binding and/or protein dimerization, and attenuates the negative regulation of circSNX5 formation by STAU1. This supports the hypothesis of STAU1’s preference for RNA duplex recognition [40, 48], and suggests that such regulation might be contingent upon the architectural formation of sequence-specific duplexes and/or tertiary RNA–RNA interactions. These insights not only broaden our understanding of circRNA biogenesis but also underscore the nuanced and multifaceted nature of RBP involvement in circRNA regulation.

The functional regulation of circRNAs in the cells is intricately linked to their spatiotemporal distribution, a factor that, when disrupted, can lead to diseases and abnormalities [49, 50]. Our study unveils an intriguing aspect of this phenomenon by identifying distinct distributions of the precursor and mature forms of circSNX5 RNA. This discovery points to a sophisticated mechanism that finely tunes the functionality of circSNX5, governing its physiological regulation and underscoring a predominant mechanism that determines the active or inactive state of the circRNome in organisms. We have further elucidated that the STAU1 protein plays a critical role in this process. It negatively regulates the sequential back-splicing of precursor circSNX5 RNA, thereby impacting both the expression levels and the nuclear-cytosolic transport of mature circSNX5 RNA. This finding reinforces the pivotal role of STAU1 in controlling the distribution of target transcripts [40] and extends its known functions to include involvement in mRNA processing and circRNA formation. Supporting this, our RNA-sequencing analysis following STAU1 knockdown revealed a notable increase in upregulated circRNAs compared to control cells (Fig. S16), highlighting the functional impact of STAU1 on circRNA formation.

Intriguingly, treatments with the HDAC1 inhibitor Trichostatin A (TSA) and the ER stress inducer Thapsigargin (Tg) significantly reduced the expression of the circSNX5 precursor transcript (Fig. S17). The observation links circRNA-associated alternative splicing to chromatin remodeling mechanisms and/or proteostasis, offering new perspectives on potential therapeutic application. These insights collectively shed light on the dynamic nature of transcription mechanisms, particularly the interplay among chromatin structural remodeling [51, 52], RBP modulation [53], and RNA polymerase II elongation [54]. They add another layer to our understanding of transcriptome processing, specifically alternative splicing, and help to contextualize the complexity and diversity inherent in this process. In doing so, they provide a comprehensive framework to interpret and connect our findings within the broader landscape of transcriptomic regulation.

### Supplementary information

Supplemental information

Supplemental Table S2

## Data Availability

The RNA-sequencing data in this study were deposited at NCBI Gene Expression Omnibus (GEO) database under the accession code GSE213634 and GSE214084. The original blots were included in the last figure of supplemental information.
