# Supplementary material for: Noncanonical formation of SNX5 gene-derived circular RNA regulates cancer growth
Source: Cell Death Dis. 2024 Aug 18;15(8):599. doi: 10.1038/s41419-024-06980-4 (PMC11330969; doi:10.1038/s41419-024-06980-4)

Noncanonical Formation of SNX5 Gene-Derived Circular RNA Regulates Cancer Growth

Yi-Tung Chen[1,2], Hui-Ju Tsai[3], Chia-Hua Kan[2], Chung-Pei Ma[2], Hui-Wen Chen[2,4], Ian Yi-Feng Chang[1,5], Hsuan Liu[1,4,6,7], Chih-Ching Wu[3], Wei-Yun Chu[4], Ya-Chun Wu[8], Kai-Ping Chang[1,9], Jau-Song Yu[1,4,6], and Bertrand Chin-Ming Tan[2,4,5,10,*]

[1]Molecular Medicine Research Center, Chang Gung University, Taoyuan 333, Taiwan
[2]Department of Biomedical Sciences, College of Medicine, Chang Gung University, Taoyuan 333, Taiwan
[3]Department of Medical Biotechnology and Laboratory Science, College of Medicine, Chang Gung University, Taoyuan 333, Taiwan
[4]Graduate Institute of Biomedical Sciences, College of Medicine, Chang Gung University, Taoyuan 333, Taiwan
[5]Department of Neurosurgery, Lin-Kou Medical Center, Chang Gung Memorial Hospital, Taoyuan 333, Taiwan
[6]Department of Cell and Molecular Biology, College of Medicine, Chang Gung University, Taoyuan 333, Taiwan
[7]Division of Colon and Rectal Surgery, Lin-Kou Medical Center, Chang Gung Memorial Hospital, Taoyuan 333, Taiwan
[8]Asia American International Academy, Taiwan
[9]Department of Otolaryngology-Head & Neck Surgery, Lin-Kou Medical Center, Chang Gung Memorial Hospital, Taoyuan 333, Taiwan
[10]Research Center for Emerging Viral Infections, Chang Gung University, Taoyuan 333, Taiwan

*Correspondence: btan@mail.cgu.edu.tw; Tel.: +886-3-211-8800; Fax: +866-3-211-8700

Supplemental Information

Materials and Methods

Cell Culture

OECM1 cells were cultured in high-glucose Dulbecco's modified Eagle's medium containing 1× Non-Essential Amino Acid (NEAA) and 1 mM sodium pyruvate. HepG2, Huh7, PLC/PRF/5, and MDA-MB-231 cells were cultured in high-glucose Dulbecco's modified Eagle's medium containing 2 mM L-Glutamine and 1 x NEAA. MCF7 cells were cultured in Minimum Essential Media containing 1 x NEAA and 1 mM sodium pyruvate. All culture media were supplemented with 10% heat-inactivated fetal bovine serum and 1 U/ml penicillin-streptomycin. All media and reagents were obtained from Thermo Fisher Scientific. The cells were incubated at 37°C with 5% CO2 in a humidified incubator.

Isolation of ribosome nascent chain-mRNA (RNC-mRNA) complex

Experimental procedures of RNC-mRNA complex extraction were performed based on the previous work [1]. Briefly, cells were treated with 100 mg/ml cycloheximide for 15 min, collected, and separated into nuclear and cytosolic fractions. The cytosolic fraction was subjected to RNC-mRNA complex isolation by centrifuging at 185,000 × g for 5 h at 4°C with sucrose solution. Sucrose solution contained 30% sucrose, ribosome buffer [20 mM HEPES/KOH (pH 7.4), 15 mM $MgCl_2$, and 200 mM KCl], 2 mM dithiothreitol, and 100 mg/ml cycloheximide. The sediment fraction was washed twice by buffer, and TRIzol reagent was used to isolate RNA. Extracted RNA was reverse transcribed into cDNA samples, and target gene expression in RNC fraction was quantified by RT-qPCR assay. ACTIN and 7SL expression served as the positive and negative control for this assay, respectively.

Pull-down assay with biotinylated probes

Pull-down assay was performed as previously described [2], using probes synthesized by MDBio. The probe sequences are listed in **Supplemental Table S1**. In brief, SAS cells were collected, lysed, and quantified. The probes were incubated with Dynabeads™ MyOne™ Streptavidin T1 magnetic beads (Thermo Fisher Scientific) to generate probe-conjugated beads for 2 h at 25°C. The cell lysates were then incubated with the control probe or circSNX5-specific probe overnight at 4°C. Subsequently, complexes were washed and subjected to RNA extraction using the RNeasy MinElute Cleanup Kit (QIAGEN). The isolated RNA was transcribed into cDNAs and analyzed by RT-qPCR. Additionally, biotinylated miR-323 mimics were synthesized and delivered into cells, and the cell lysate was harvested and subjected to the pull-down assay as described above.

Table S1. List of primers used in this study.

Primers	Sequence (5' to 3')	Application	Size
PCR-circSNX5-Forward	CGTTTCAGAGCCCAGAGTTT	End-point PCR	177 bp
PCR circSNX5-Reverse	AAGCGAGGGATCAACATTCA		
PCR-ACTIN-Forward	TGGGACGACATGGAGAAAA	End-point PCR	563 bp
PCR-ACTIN-Reverse	AAGGAAGGCTGGAAGAGTGC		
PCR-circMMP12-Forward	CAGCCATGCCTGTGTTAATC	End-point PCR	214 bp
PCR-circMMP12-Reverse	GGAATCCTAGCCCATGCTTT		
PCR-circBNC2-Forward	TGAAAGAGATGCACGTCTGC	End-point PCR	209 bp
PCR-circBNC2-Reverse	CTGCCCTTCTTTCTCCTGAA		
circSNX5-Con-Forward	CGTTTCAGAGCCCAGAGTTT	Convergent PCR	253 bp
circSNX5-Con-Reverse	CACATACTTTCCACTTGACAG		
circSNX5-Div-Forward	GTCAAATTTACAGTGCACAC	Divergent PCR	177 bp
circSNX5-Div-Reverse	AAGCGAGGGATCAACATTCA		
SNX5-exon 2-Forward	GTCAAATTTACAGTGCACAC	circRNA map	179/1626 bp
SNX5-exon 2-Reverse	AAGCGAGGGATCAACATTCA		
circSNX5-Forward	CGTTTCAGAGCCCAGAGTTT	RT-qPCR	177 bp
circSNX5-Reverse	AAGCGAGGGATCAACATTCA		
circSNX5-precursor-Forward	GGTGTGAACAGTGTAATGAC	RT-qPCR	152 bp
circSNX5-precursor-Reverse	CTGTAATCCCAGCACTTTGG		
ACTIN-Forward	CCAACCGCGAGAAGATGA	RT-qPCR	97 bp
ACTIN-Reverse	CCAGAGGCGTACAGGGATAG		
EGR1-Forward	CAGCAGCAGCACCTTCAACCCT	RT-qPCR	137 bp
EGR1-Reverse	GAGTGGTTTGGCTGGGGTAACT		
U48-Forward	AGTGATGATGACCCCAGGTA	RT-qPCR	63 bp
U48-Reverse	GGTCAGAGCGCTGCGGTGAT		
7SL-Forward	CGCACTAAGTTCGGCATCAAT	RT-qPCR	108 bp
7SL-Reverse	GAGTTTTGACCTGCTCCGTTT		
SNX5-Proximal-Forward	CGTTTCAGAGCCCAGAGTTT	RT-qPCR	170 bp

SNX5-Proximal-Reverse	AGTTCTTGTTTCATCTTGGC		
SNX5-Distal-Forward	GATGTTTGGTGGCTTCTTCA	RT-qPCR	202 bp
SNX5-Distal-Reverse	TGCGGTGTGGATATAGTCAT		
STAU1-Forward	CACGAGACCCTCTGAGCAACT	RT-qPCR	119 bp
STAU1-Reverse	GAGAGGAGCAATTGATAAGAG		
UPF2-Forward	GGAGAAAACACCTAACATCACCA	RT-qPCR	86 bp
UPF2-Reverse	CCTTGTCAGTGAAAATCCCAACT		
Control siRNA-Forward	GAUCAUACGUGCGAUCAGA	RNAi	-
Control siRNA-Reverse	UCUGAUCGCACGUAUGAUC		
UPF2 siRNA-Forward	CGCGAGGGUUAAUCUUCUCUU	RNAi	-
UPF2 siRNA-Reverse	AAGAGAAGAUUAACCCUCGCG		
sh-circSNX5-1-Forward	CCGGGGGCTTATTCTGAGATCTGTACTCGAGTACAGATCTCAGAATAAGCCCTTTTT	Construction	-
sh-circSNX5-1-Reverse	AATTAAAAAGGGCTTATTCTGAGATCTGTACTCGAGTACAGATCTCAGAATAAGCCC		
sh-circSNX5-2-Forward	CCGGGCTGGGCTTATTCTGAGATCTCTCGAGAGATCTCAGAATAAGCCCAGCTTTTT	Construction	-
sh-circSNX5-2-Reverse	AATTAAAAAGCTGGGCTTATTCTGAGATCTCTCGAGAGATCTCAGAATAAGCCCAGC		
sh-STAU1-Forward	CCGGGAGGTGAATGGAAGAGAATCCCTCGAGGGATTCTCTTCCATTCACCTCTTTTT	Construction	-
sh-STAU1-Reverse	AATTAAAAAGAGGTGAATGGAAGAGAATCCCTCGAGGGATTCTCTTCCATTCACCTC		
nat circSNX5-Forward	GTGAAGAATGGGTTTCCCCTC	Construction	2876 bp
nat circSNX5-Reverse	CTGTTCCTGCCGCCAAGAAAT		
circSNX5-AluY del-Forward	TCAACTGACATTTCTAAATGATC	Construction	-
circSNX5-AluSx3 Del-Reverse	GCCTCCTTCAGCAACTTTTGAGA	Construction	-

circSNX5-AluSX del-Forward	GCCCTGGGCTGGCATCCAAGGGAAGTTTGTC	Construction	-
circSNX5-AluSX del-Reverse	CCTTGGATGCCAGCCCAGGGCTCTGCCACA		
circSNX5-AluSZ6 del-Forward	TCAGACTTACAGAATTAGGGTTGTGGCTTA	Construction	-
circSNX5-AluSZ6 del-Reverse	CCCTAATTCTGTAAGTCTGAATTTACCCCC		
circSNX5-Polypyrimidine-Forward	AGCCAGGGACGTGGGGGTAAATTCAGACTT	Construction	-
circSNX5-Polypyrimidine-Reverse	TACCCCCCACGTCCCTGGCTTCCTTCCTTTT		
circMMP12-Forward	TCTAGAAGGAAGAATTGTTCA	Construction	1671 bp
circMMP12-Reverse	CTCGAGGCAACATAGCAAGATCCT		
circBNC2 exon-Forward	GATGCTGCTGGCAAGGTGCT	Construction	1970 bp
circBNC2 exon-Reverse	CTGTCTCGGCTTCGGCGAGAG		
STAU1-Forward	TCTAGAATGAAACTTGGAAAAAAACCAATGTA	Construction	1491 bp
STAU1-Reverse	GATATCTCAGCACCTCCCACACACAGAC		
STAU1-RBD4 mutant-Forward	GCAGAAGGAACGGGCACCAACGCTGCTGTGGCCAAGCGCAATGCA	Construction	-
STAU1-RBD4 mutant-Reverse	TGCATTGCGCTTGGCCACAGCAGCGTTGGTGCCCGTTCCTTCTGC	Construction	-
shSTAU1-resistant-Forward	GCCCCTGCCAGAGAGGCTGGAAGTAAACGGCCGCGAGTCA	Construction	-
shSTAU1-resistant-Reverse	GATTTATTGAGATTTTCTTCTTCTGACTCGCGGCCGTTTACTTC	Construction	-
Control-mimic-Forward	UUCUCCGAACGUGUCACGUTT	miRNA	-
Control-mimic-Reverse	ACGUGACACGUUCGGAGAATT		
miR-323a-5p-mimic-Forward	AGGUGGUCCGUGGCGCGUUCGC	miRNA	-
miR-323a-5p-mimic-Reverse	GAACGCGCCACGGACCACCUUU		
U6-Forward	CTCGCTTCGGCAGCACA	RT-qPCR	-
U6-Reverse	AACGCTTCACGAATTTGCGT		

miR-323a-5p-Forward	CGGCGGAGGTGGTCCGTGGCGC G	RT-qPCR	-
Universal Reverse	CAACTGGTGTCGTGGAGTCGG		
ADAM10-UTR-Forward	AGACCTCCACAGCCCATTCA	Construction	885 bp
ADAM10-UTR-Reverse	GAACTCTGAAGTAGTATGTG		
ADAM10-UTR mutant-Forward	GGAGGCTCAGTTGTTTCAACATT GGAGACATCACTT	Construction	-
ADAM10-UTR mutant-Reverse	TCCAATGTTGAAACAACTGAGCC TCCTAGCCTTGATTGG		
Control probe (-biotinylated)	ACTATACGACACAGCACGTAATC AG	Pull-down assay	-
circSNX5 probe (-biotinylated)	ATACAGATCTCAGAATAAGCCCA GC		
miR-323a-5p mimic (-biotinylated)	AGGUGGUCCGUGGCGCGUUCGC		

Table S2. Proteomic analyses of circSNX5 knockdown cells

Please find the attached excel file.

Reference

1. Chen, Y.T., et al., *Tumor-associated intronic editing of HNRPLL generates a novel splicing variant linked to cell proliferation.* J Biol Chem, 2018. **293**(26): p. 10158-10171.
2. Li, Y., et al., *CircHIPK3 sponges miR-558 to suppress heparanase expression in bladder cancer cells.* EMBO Rep, 2017. **18**(9): p. 1646-1659.

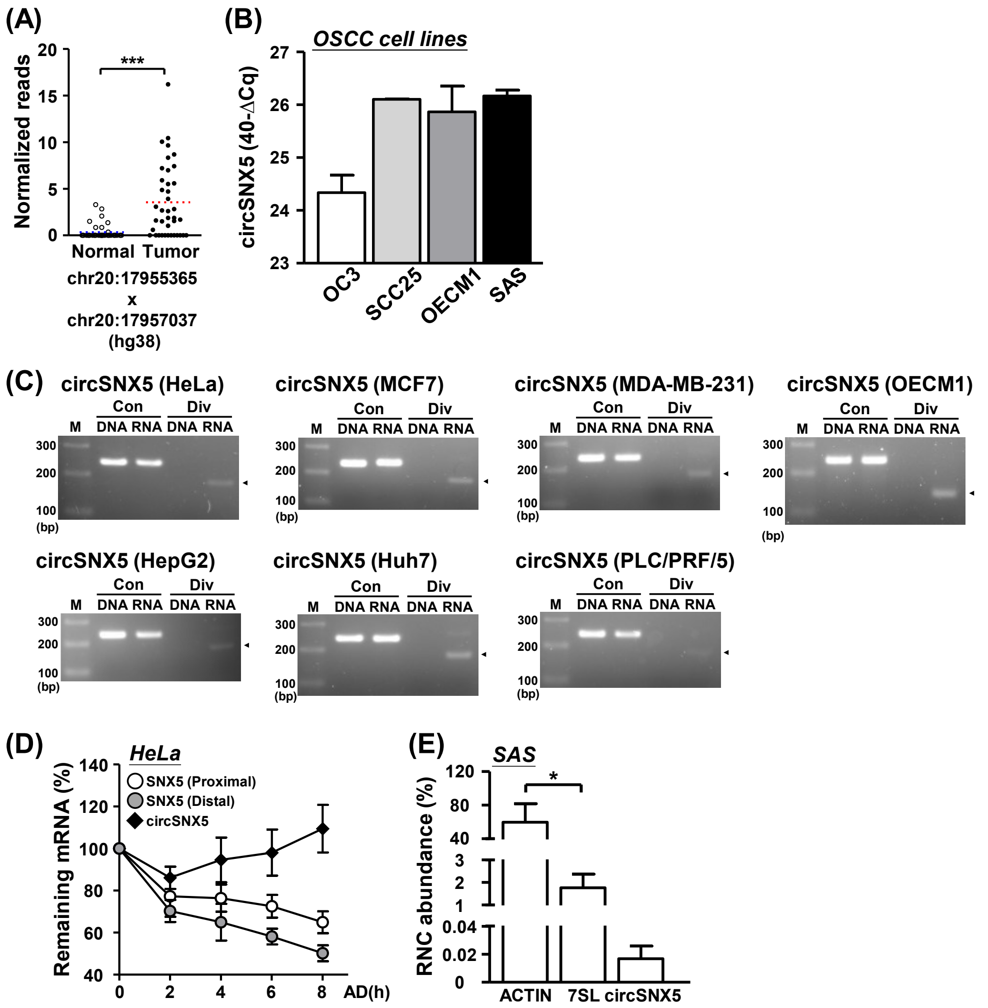

Figure S1. CircSNX5 RNA was expressed in cancer cell lines. (A) RNA-sequencing read counts corresponding to the indicated back-splicing event, with the annotated chromosomal location shown at the bottom. (B) CircSNX5 RNA expression of oral cancer cell lines were analyzed by RT-qPCR experiment and presented by the ΔCq method. (C) Genomic DNA (DNA) and reverse-transcribed cDNA (RNA) of the indicated cells were subjected to PCR assays with convergent (Con) and divergent (Div) primers, and the resulting PCR products were analyzed by gel electrophoresis. (D) HeLa cells were treated with actinomycin D (AD) for the indicated time lengths, and total RNA of treated cells at time-points were collected and subjected to RT-qPCR assays. RNA turnover rate was measured by normalization of RNA abundance to the initial time-point and plotted for individual gene. (E) RNC-mRNA fraction was isolated from cytosolic fraction of the cells, and the indicated gene expression in fractions were determined by RT-qPCR assay. Indicated gene abundance in RNC fraction was calculated by ΔCq method, and normalized to the values of cytosolic fractions.

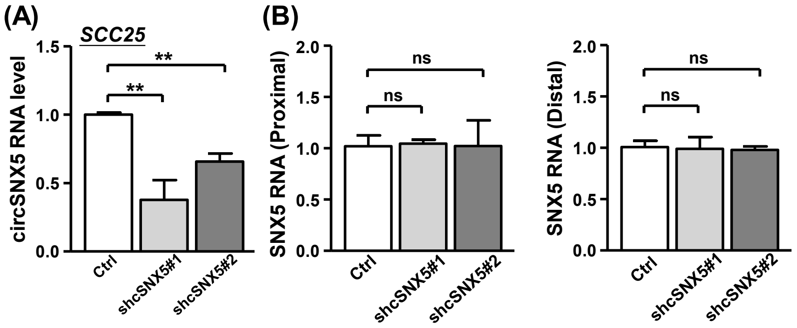

Figure S2. Parental SNX5 mRNA level remains invariable at circSNX5 knockdown. (A) CircSNX5 RNA expression of control and knockdown SCC25 cells was analyzed by RT-qPCR experiment. (B) Parental SNX5 gene expression of control and circSNX5 knockdown cells were detected by RT-qPCR assays, and the proximal and distal region primers corresponding to back-splicing junction site were used.

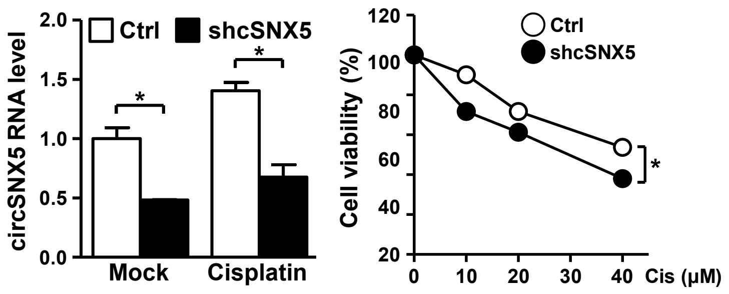

Figure S3. CircSNX5 knockdown enhances the cisplatin sensitivity of cancer cells. Control and circSNX5 knockdown cells were treated with the indicated concentration of cisplatin, and the gene expression and cell survival of the treated cells was analyzed by RT-qPCR and MTT assays.

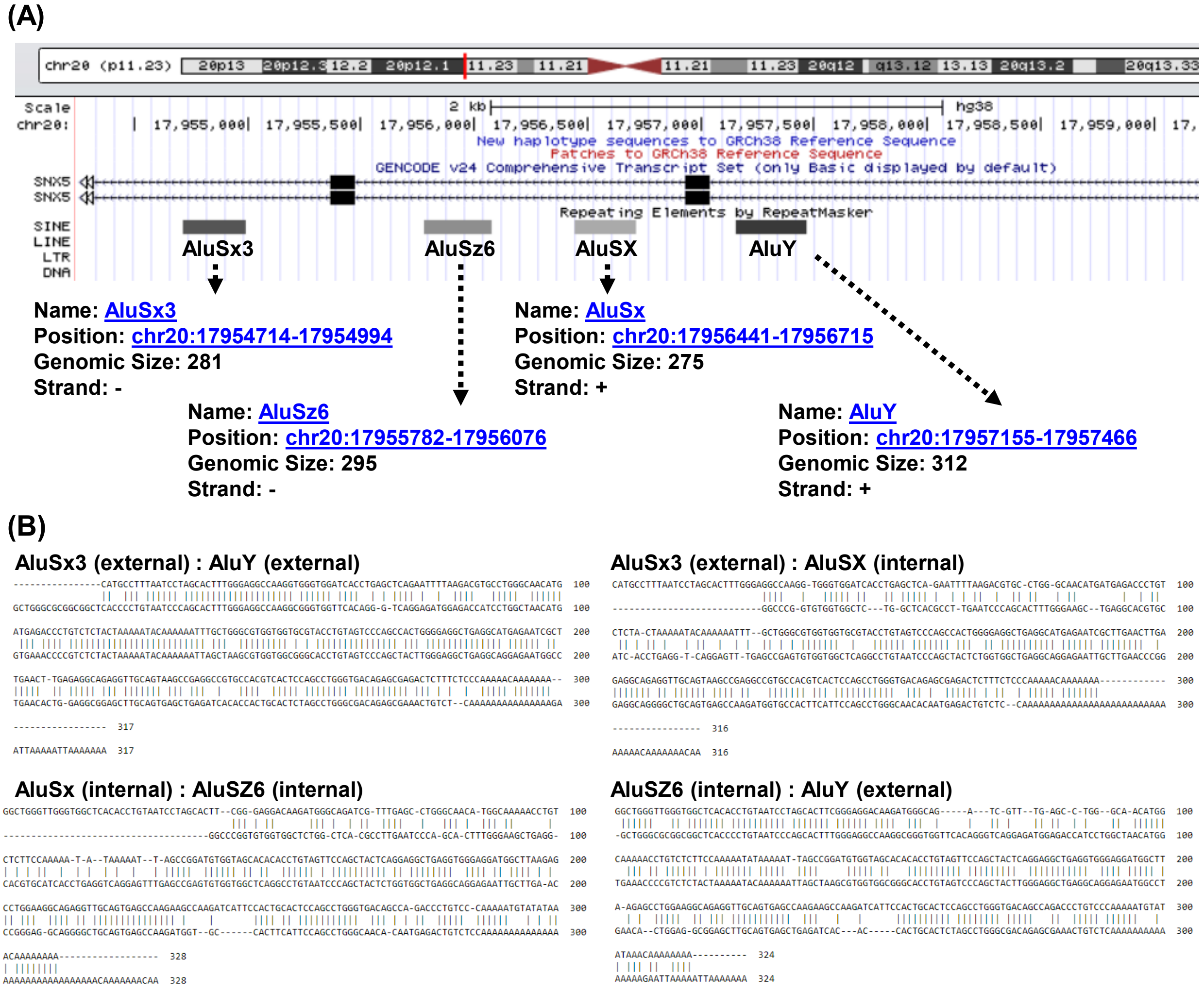

Figure S4. The master repeats involve with the back-splicing formation on SNX5 gene. (A) Neighboring master repeats to the circSNX5 gene were uncovered by UCSC Genome Browser, and the attributes of repeats were noted in the lower panel. (B) Illustration for the sequence alignments of the indicated master repeats.

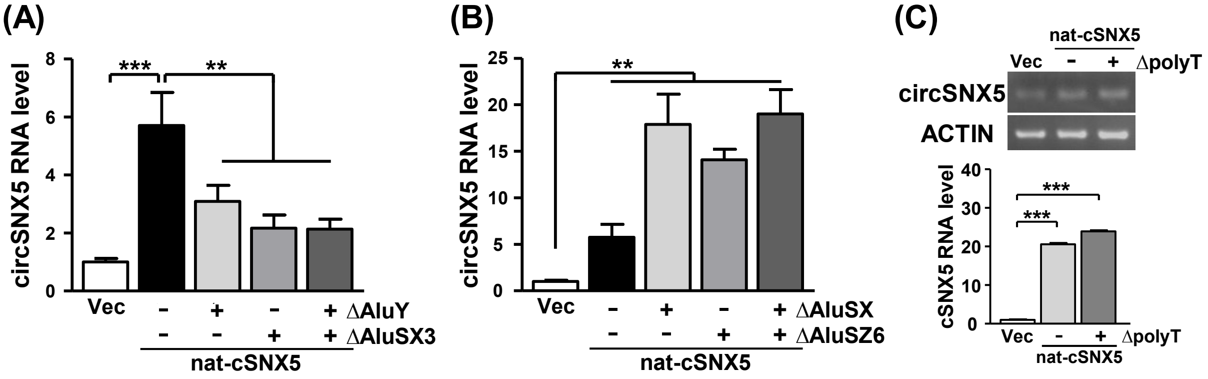

Figure S5. Cis-acting motifs mediate the alternative back-splicing of SNX5 RNA. (A and B) CircSNX5 RNA expression of the transfectants in **Fig 3B** were measured by RT-qPC. (C) Polypyrimidine (polyT) removal of circSNX5 expression construct was established and transfected into cells, and the circSNX5 expression in the transfectants was analyzed by PCR assays. ACTIN expression served as the loading control.

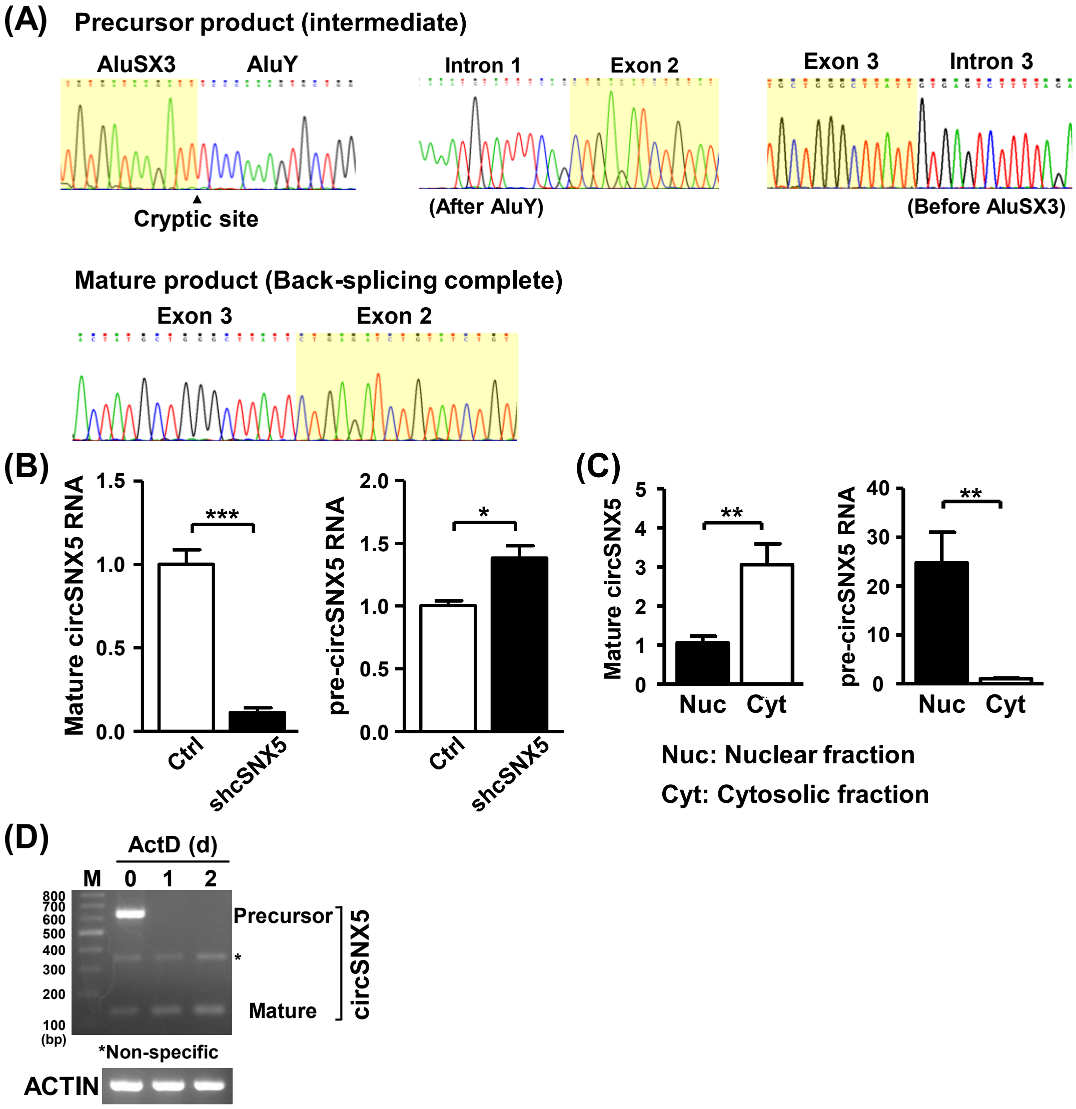

Figure S6. Noncanonical sequential back-splicing takes places in the circSNX5 formation. (A) PCR products in **Fig 3D** were purified and analyzed by the Sanger method, and the results were shown in sequence histograms. The region on the SNX5 gene was annotated and highlighted. (B) Mature and precursor circSNX5 RNA expression of the indicated cells in **Fig 3E** were analyzed by RT-qPCR. (C) Indicated circSNX5 RNA levels of the subcellular fractions in **Fig 3F** were measured by RT-qPCR experiments. (D) Cells were treated with actinomycin (ActD) at the indicated time points, and the total RNA of treated cells was harvested and transferred into cDNA. CircSNX5 PCR assays of cDNA samples were performed and visualized by gel electrophoresis, and the PCR amplicons were annotated. ACTIN expression served as the loading control.

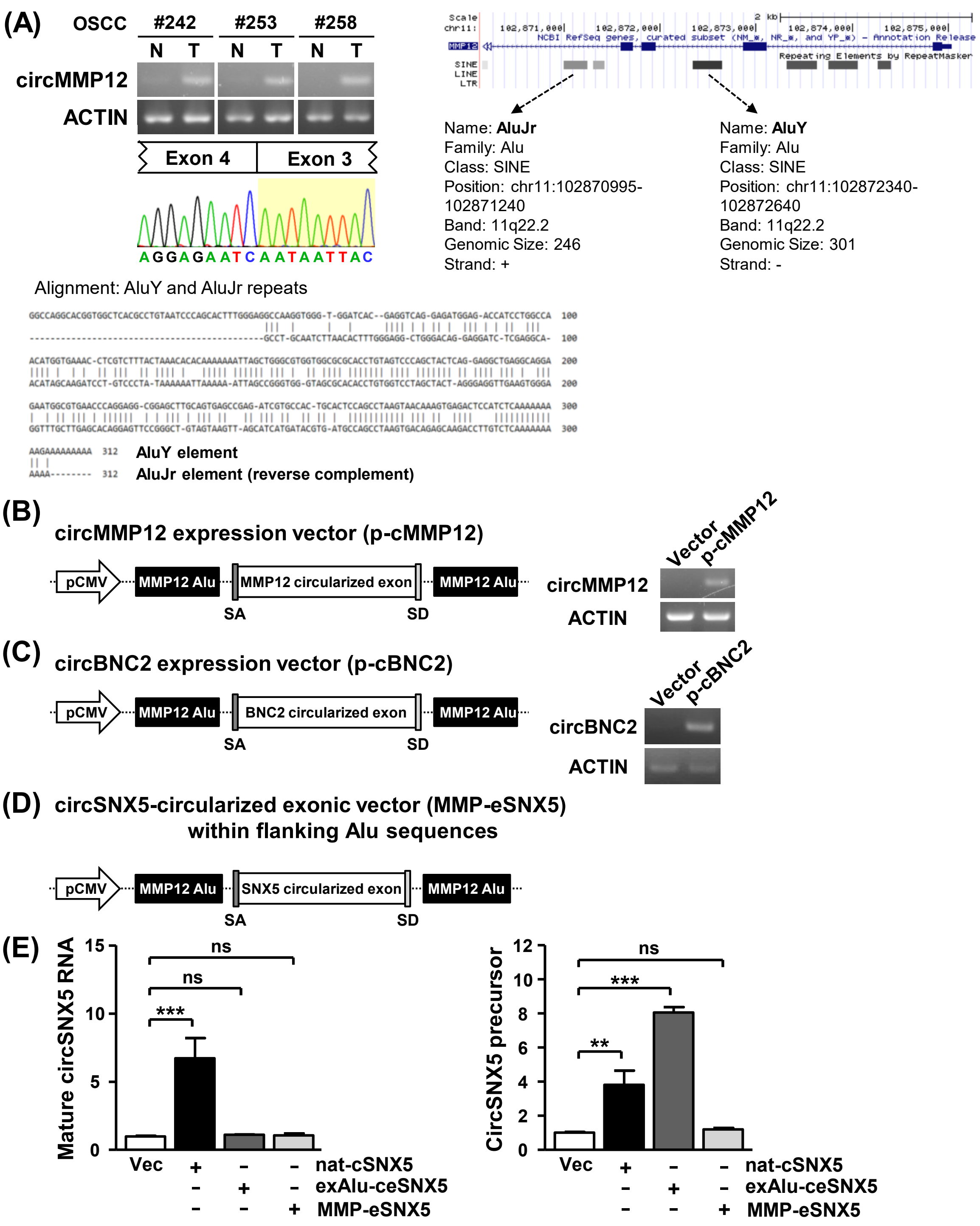

Figure S7. Activation of back-splicing by Alu elements derived from the MMP12 gene. (A) CircMMP12 expression in the paired OSCC samples was detected by end-point PCR assays, and PCR products were visualized by gel electrophoresis and further analyzed by the Sanger method (upper left panel). Back-splicing junctions of circMMP12 RNA were highlighted in the sequence histograms. Spanning master repeats of MMP12 gene were revealed by UCSC genome browser (upper right panel), and the sequence alignment of the two repeats was performed and shown in the lower panel. (B) Schematic illustration for circMMP12 expression vector construction (p-cMMP12), and the expression efficacy of plasmid was analyzed by PCR assays. ACTIN expression served as the loading control. (C)

CircBNC2 construct (p-cBNC2) was established and delivered into cells, and the expression efficacy of the construct vector was assessed by PCR. ACTIN expression served as the internal control. (D) CircSNX5 construct (MMP-eSNX5) was established by sub-cloning the circularized exons and the flanking MMP12 Alu repeats of circSNX5 RNA into the vector backbone. (E) Mature and precursor circSNX5 RNA levels of the transfectants in **Fig 3G** were measured by RT-qPCR assays.

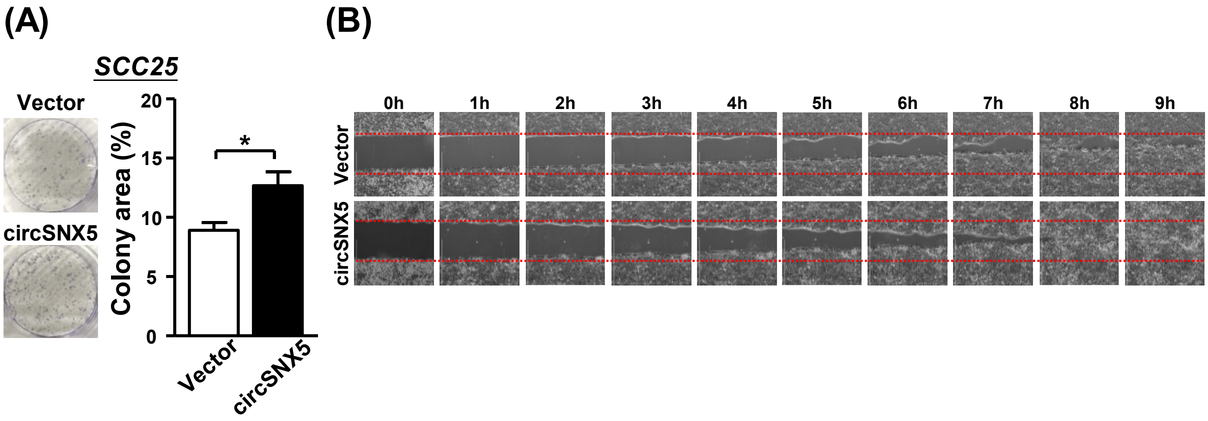

Figure S8. CircSNX5 overexpression enhances the colony formation and cell migration abilities of oral cancer cell lines. (A) Colony formation assays of control and circSNX5 overexpression SCC25 cells were performed and visualized by crystal violet staining, and the quantification of colony forming was depicted in the bar graph. (B) The wound healing migration assay was performed to assess the migratory ability of SAS cells upon circSNX5 overexpression. Representative images of the culture at the indicated time points after scratching are shown.

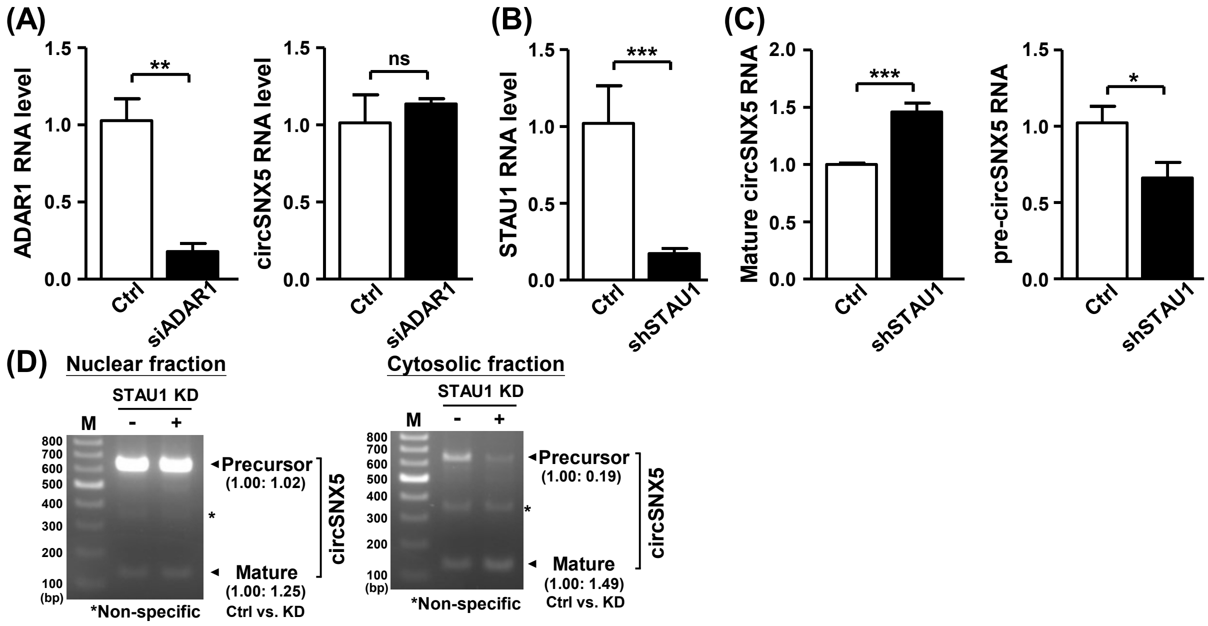

Figure S9. STAU1 knockdown facilitates the circSNX5 formation. (A) Control and ADAR1-specific siRNAs were transfected into cells, and the indicated gene expression of the transfectants was analyzed by RT-qPCR assays. (B) STAU1 RNA levels of the indicated cells were measured by RT-qPCR. (C) Precursor and mature circSNX5 RNA levels of the transfectants in **Fig 5B** were measured by RT-qPCR. (D) CircSNX5 PCR assays of the indicated samples were performed, and the resulting PCR products were resolved by gel electrophoresis. PCR amplicons were noted and quantified.

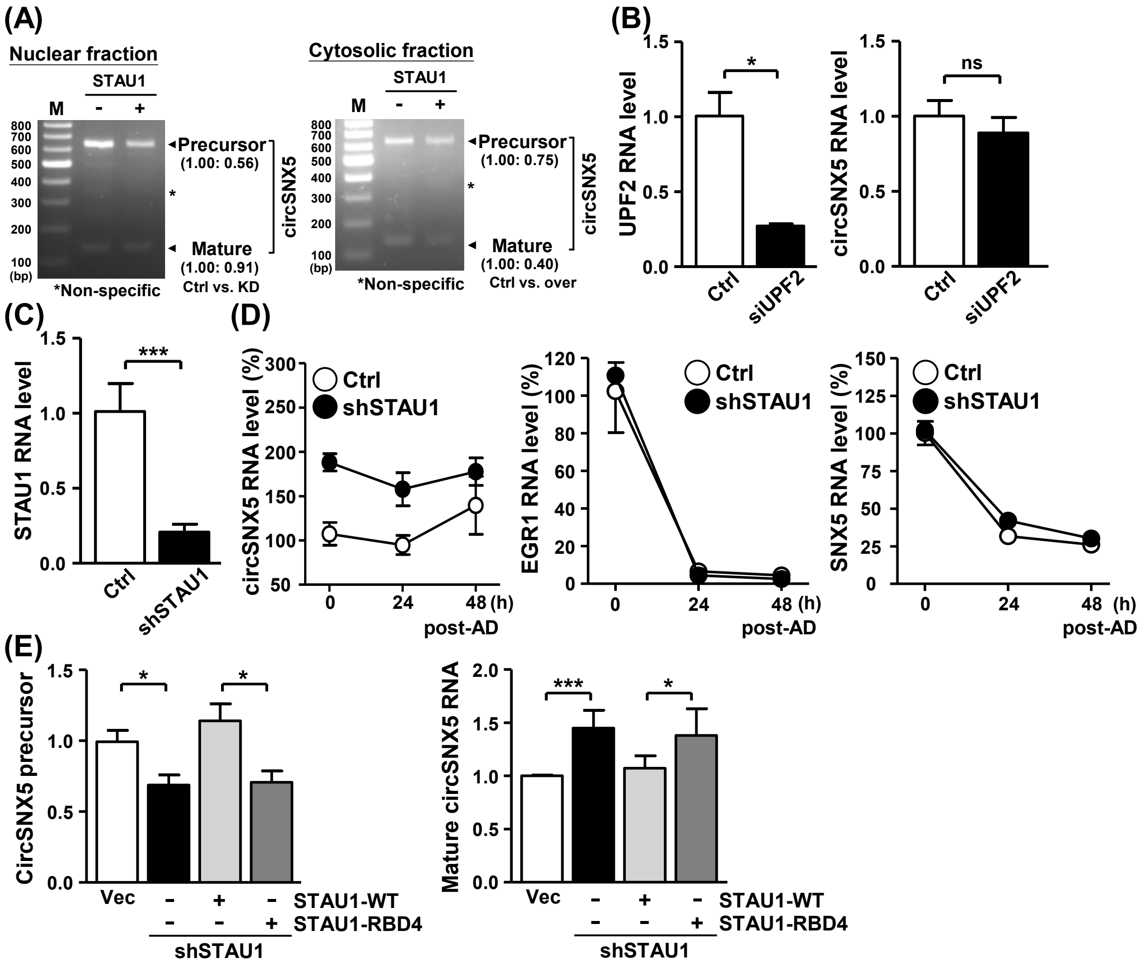

Figure S10. STAU1 protein negatively regulates the back-splicing of circSNX5 formation. (A) CircSNX5 PCR assays of the fractions from control and STAU1 overexpression cells were performed, and the resulting PCR products were visualized by gel electrophoresis. PCR amplicons were noted and quantified. (B) Control and UPF2-specific siRNAs were transfected into cells, and the indicated gene expression of transfectants was analyzed by RT-qPCR. (C) STAU1 RNA expression in the indicated cells was analyzed by RT-qPCR. (D) Control and STAU1 knockdown cells were treated with actinomycin D (AD) for the indicated time lengths, and the total RNA of treated cells was collected and subjected to RT-qPCR for analysis of target genes. RNA turnover rate was measured by normalization of RNA abundance relative to the initial time point and plotted. (E) CircSNX5 precursor and mature variant RNA levels of the transfectants in **Fig 5G** were analyzed by RT-qPCR.

(A)

Pathway	P-value	Count	Genes
Pathways in cancer	1.35×10^{-2}	7	NOTCH3, CDKN2B, KITLG, CDKN2A, PPARG, MITF, IL7R
Transcriptional misregulation in cancer	3.61×10^{-2}	4	IGFBP3, PPARG, MITF, ETV5
Complement and coagulation cascades	4.11×10^{-2}	3	C3, THBD, F3
ECM-receptor interaction	4.37×10^{-2}	3	FRAS1, TNC, THBS2

(B)

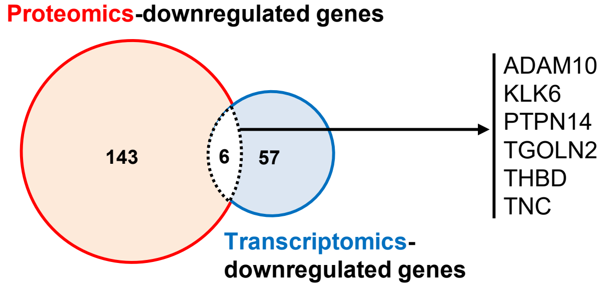

Figure S11. Omics profiles of the circSNX5 knockdown cells. (A) Differentially expressed genes revealed by the RNA-sequencing assay were subjected to pathway analysis by the DAVID bioinformatics, and the annotated pathways were ranked and shown. (B) The intersection of the differentially expressed gene sets from the proteomic and transcriptomic profiling was performed to explore the downstream target gene network.

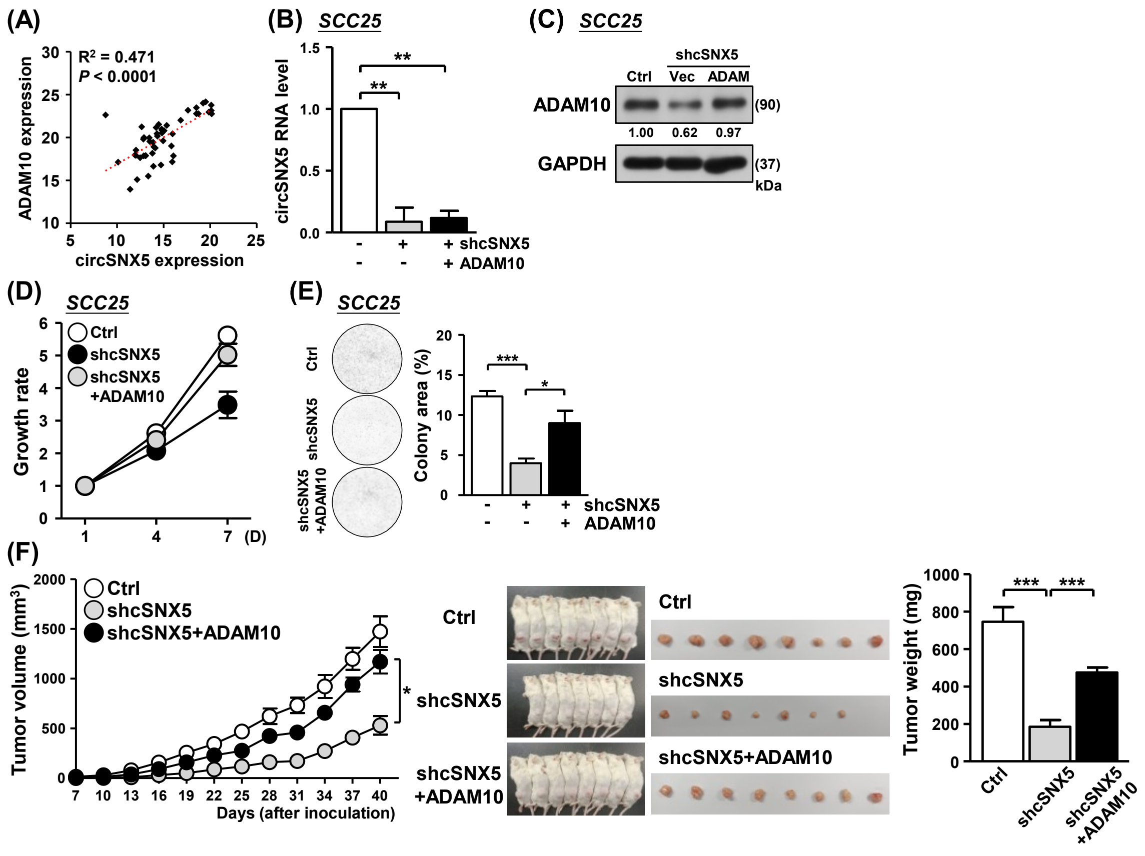

Figure S12. Ectopic ADAM10 expression moderates the inhibited phenotypes by circSNX5 knockdown. (A) RT-qPCR results for circSNX5 and ADAM10 RNA expression in the OSCC cohort were subjected to a co-expression analysis (n = 48), and the R-squared value and P-value were calculated and noted. (B) Indicated RNA expression in the transfected SCC25 cells was determined by RT-qPCR. (C) ADAM10 protein expression in the indicated transfected SCC25 cells was measured by Western blot. GAPDH expression served as the loading control. (D and E) Cell proliferation and colony formation ability of the indicated transfectants of SCC25 cells were analyzed by MTT assay and crystal violet staining, respectively. Colony images and quantified results are shown. (F) The mouse xenograft experiment was performed by inoculating the indicated transfected SAS cell lines. Tumors formed at the indicated time points were dissected and measured for volume (left panel). The photographs of mice bearing tumors and the weight of resected tumors are shown.

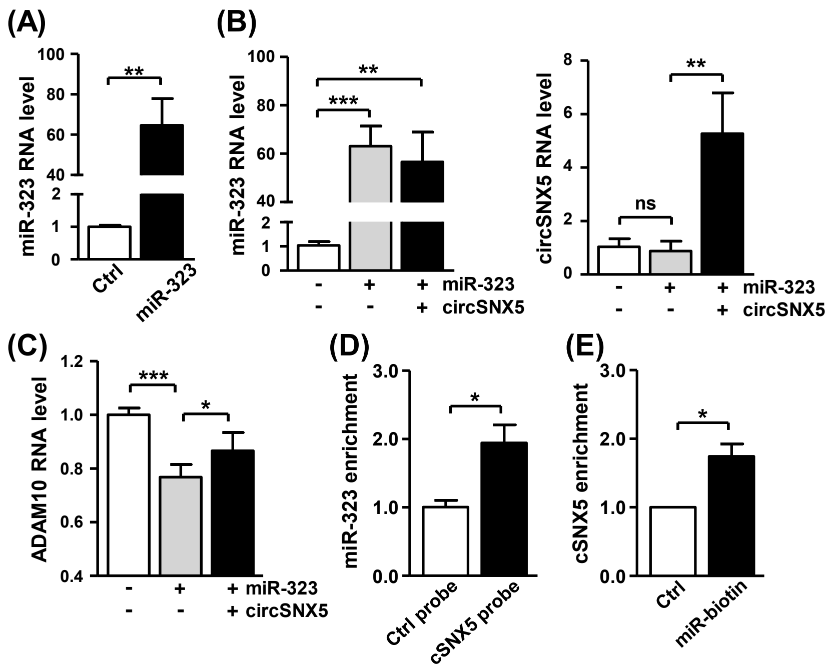

Figure S13. CircSNX5 RNA functions as a miRNA sponge to regulate oral cancer progression. (A) MiR-323 RNA levels in the transfectants in **Fig 6H** were collected and subjected to RT-qPCR. (B) MiR-323 and circSNX5 RNA expression in the transfectants in **Fig 6I** were determined by RT-qPCR. (C) ADAM10 RNA expression in the indicated transfected cells was analyzed by RT-qPC. (D) Pull-down assay was performed with the biotinylated circSNX5-specific probe and control oligo, and the enrichment of miR-323 in the pull-down complex was analyzed by RT-qPCR. (E) Biotin-labeled miR-323 was delivered into SAS cells, and the transfected cells were subjected to a pull-down assay. The enrichment of circSNX5 RNA in the complex was analyzed by RT-qPCR.

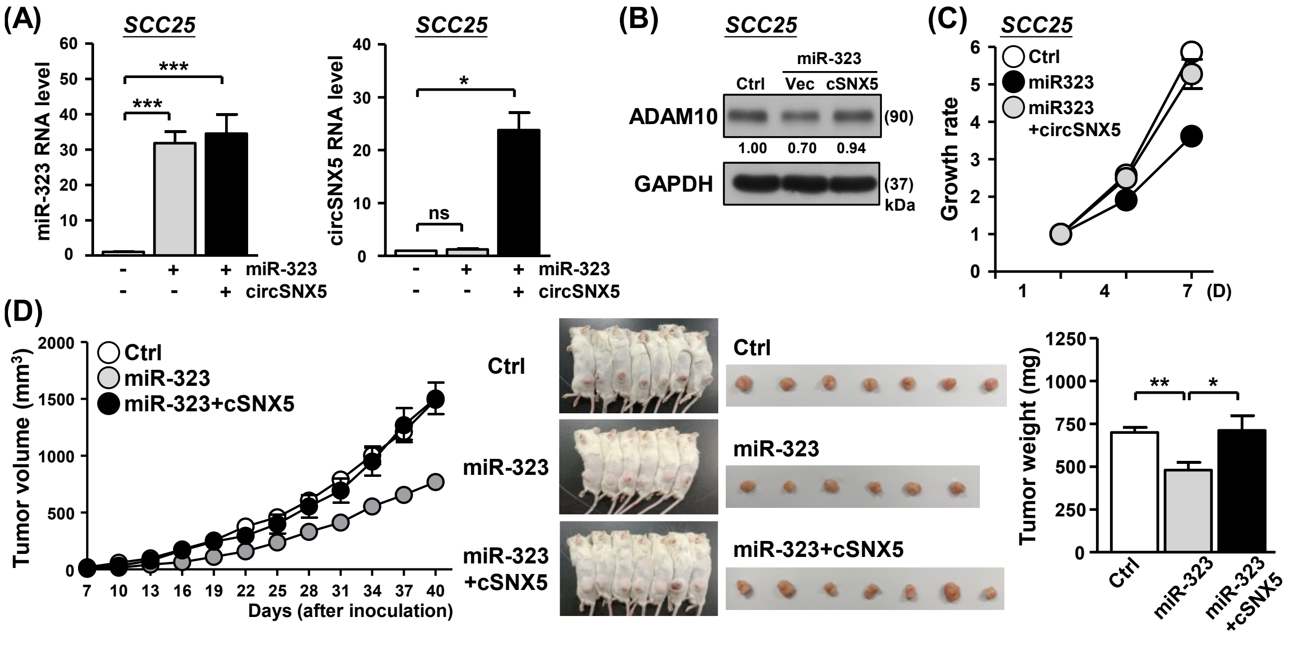

Figure S14. CircSNX5 expression antagonizes miR-323 to regulate SCC25 cell growth. (A and B) The indicated transfectants of SCC25 cells were collected and subjected to RNA and protein expression analysis by RT-qPCR and Western blot. GAPDH expression served as the loading control for immunoblotting. (C) Cell proliferation of the transfectants in SCC25 cells was analyzed by the MTT method. (D) The mouse xenograft experiment was performed by inoculating the indicated SAS cell lines. Tumors formed at the indicated time points were dissected and measured for volume (left panel). The photographs of mice bearing tumors and the weight of resected tumors are shown.

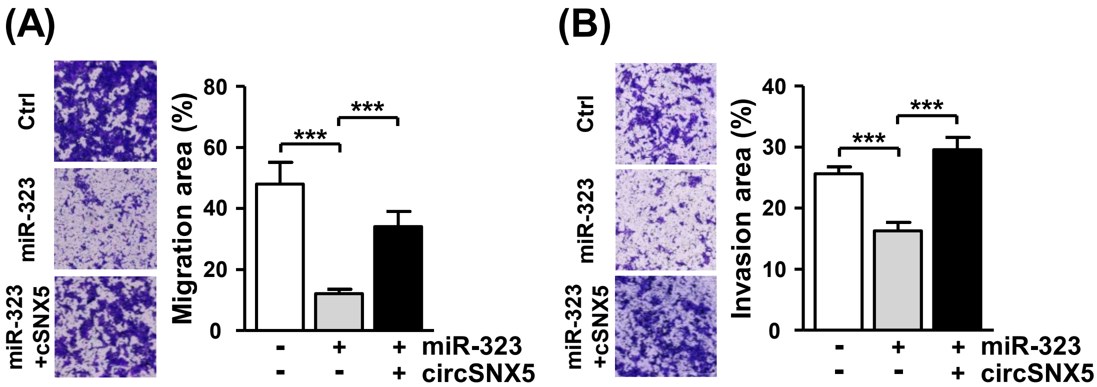

Figure S15. CircSNX5 expression neutralizes miR-323-induced downregulation of cell metastasis. (A and B) Transwell migration and invasion assays of the indicated transfectant SAS cells were performed. The representative photographs for the indicated groups were shown in the left panel, and the migration and invasion capacities were quantified and shown in bar graphs.

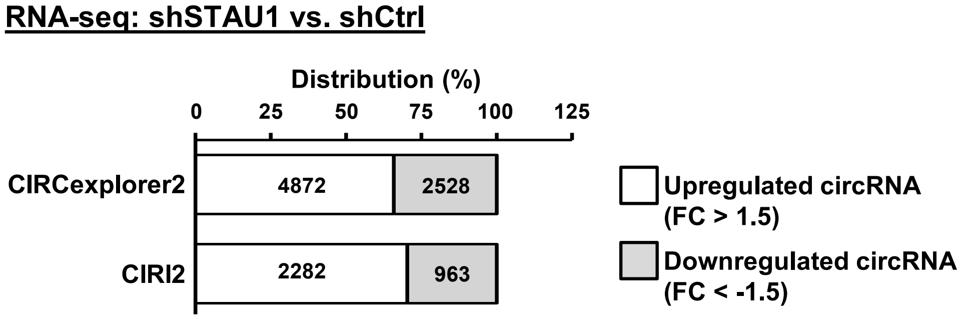

Figure S16. STAU1 knockdown leads to notably upregulation of circRNAs. Control and STAU1 knockdown cells were collected and subjected to RNA-sequencing assay, and the obtained reads were analyzed by CIRCexplorer2 and CIRI2 algorithms to call back-splicing events (circRNAs). The differentially expressed circRNAs between control and knockdown cells were filtered by the fold change more than 1.50, and the numbers of which were noted and plotted to stacked bar graphs. The proportion of upregulated and downregulated circRNAs served as the x-axis, and the used analytics were showed in y-axis.

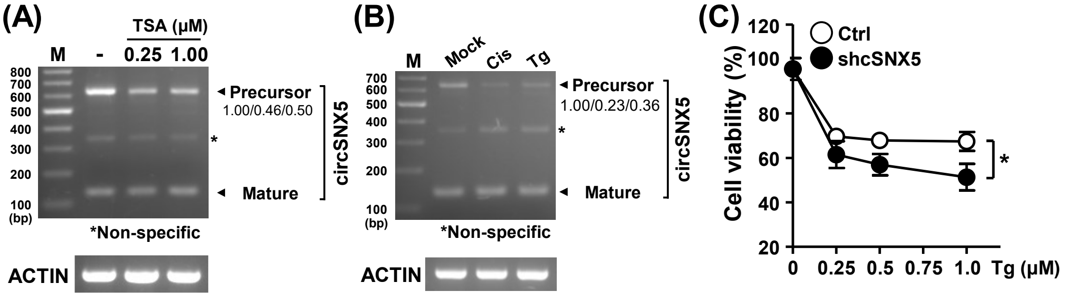

Figure S17. HDAC inhibitor and ER stress inducer downregulate circSNX5 precursor expression. (A and B) CircSNX5 PCR assays of the drug-treated samples were performed, and the resulting PCR products were visualized by gel electrophoresis. PCR amplicons were noted and quantified. ACTIN expression served as the internal control. (C) Control and circSNX5 knockdown cells were treated with the indicated concentration of Thapsigargin (Tg), and the cell viability after drug challenge was analyzed by MTT method and further plotted.

Original Western Blots

Fig 2J

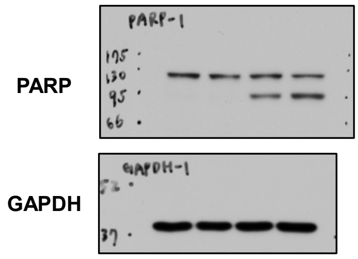

Fig 4G

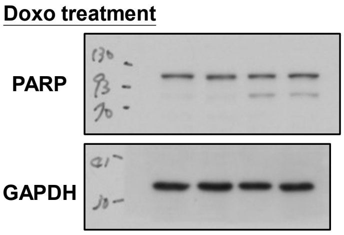

Fig 4I

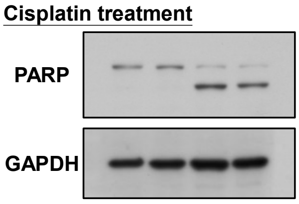

Fig 5A

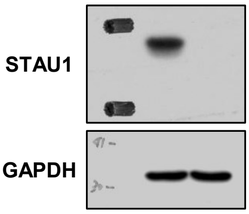

Fig 5D

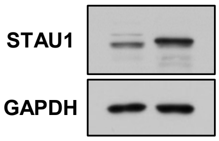

Fig 5G

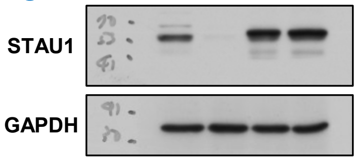

Fig 7H

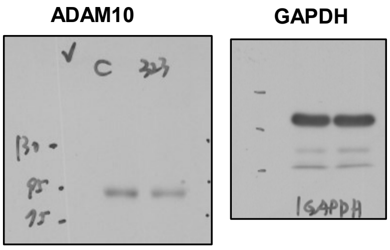

Fig 7C

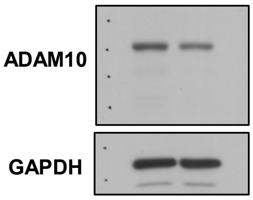

Fig 7D

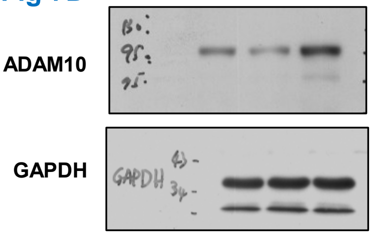

Fig 7I

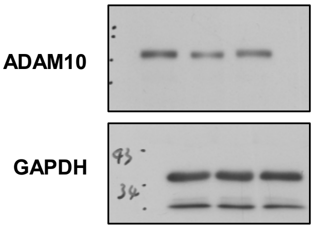

Fig S12C

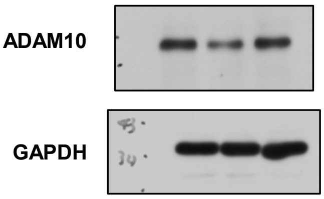

Fig S14B

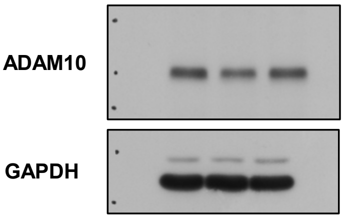

Supplement: Supplementary file 1 — Supplemental information [file 41419_2024_6980_MOESM1_ESM.pdf]
